# Programmatically activated DNA hydrogel microcapsules for precision therapy in inflammatory bowel disease

**DOI:** 10.7150/thno.111583

**Published:** 2025-05-08

**Authors:** Peifen Lu, Hongxiu Yuan, Gang Wang, Yixi Dong, Runyu Zhao, Jia Man, Jianwei Jiao, Zhaoyin Wang, Jin Jiao

**Affiliations:** 1 Shandong Cancer Hospital and Institute, School of Life Sciences, Shandong First Medical University & Shandong Academy of Medical Sciences, Jinan 250117, China; 2 Key Laboratory of High Efficiency and Clean Mechanical Manufacture of Ministry of Education, School of Mechanical Engineering, Key National Demonstration Center for Experimental Mechanical Engineering Education, Shandong University, Jinan 250061, PR China; 3 State Key Laboratory of Stem Cell and Reproductive Biology, Institute of Zoology, Chinese Academy of Sciences, Beijing 100101, China; 4 Collaborative Innovation Center of Biomedical Functional Materials and Key Laboratory of Biofunctional Materials of Jiangsu Province, School of Chemistry and Materials Science, Nanjing Normal University, Nanjing 210023, China

**Keywords:** DNA hydrogel, oral microspheres, multistage delivery microcapsules, inflammation suppression, inflammatory bowel disease.

## Abstract

**Rationale:** DNA-based nanomedicines have shown significant therapeutic potential for various diseases; however, efficiently utilizing DNA nanomedicines without chemicals or small-molecule drugs is still a major challenge.

**Methods:** In this study, we presented programmed activated DNA hydrogel microcapsules (HAMs) specifically designed for the treatment of inflammatory bowel disease (IBD). It was constructed by encapsulating a DNA hydrogel in sodium alginate microcapsules (AMs) shells. The DNA hydrogel is self-assembled from aptamer-functionalized tetrahedral DNA nanostructures (TDNs) with anti-inflammatory properties and a Y-shaped DNA scaffold in response to ATP. This design provides HAMs with characteristics of precisely targeted release and enhanced local concentrations, thus ensuring better therapeutic outcomes.

**Results:** HAMs exhibited a multistage response to intestinal fluids, a characteristic positive charge at IBD lesions, a high concentration of ATP in the inflammatory microenvironment, and high expression of the membrane protein TLR4 on immune cells, thereby enabling precisely targeted therapy for IBD. Both *in vivo* and *in vitro* studies demonstrated that this system possessed precise targeting ability and excellent stability. In a dextran sodium sulfate-induced colitis model, we demonstrated that HAMs effectively alleviate IBD by reducing the production of inflammatory cytokines, restoring the intestinal barrier, and modulating the diversity of the gut microbiota. Furthermore, no significant long-term toxicity of HAMs was detected in the treated mice.

**Conclusions:** This stable, specific, and highly biocompatible system of programmatically activated HAMs overcomes the challenges associated with developing pure DNA nanostructures for therapy and presents a promising approach for IBD treatment.

## Introduction

Inflammatory bowel disease (IBD) is a chronic and recurrent inflammatory condition of the gastrointestinal (GI) tract characterized by lifelong symptoms and frequent complications 1, 2. Compromised intestinal mucosal barrier function, host immune system responses and dysregulation of the intestinal microflora are widely considered to be associated with the progression of IBD 3. Clinically, anti-inflammatory and immunosuppressive small-molecule drugs, including systemic corticosteroids, the tumor necrosis factor-α (TNF-α) inhibitor infliximab, and amino salicylic acid, are common approaches for the treatment of IBD 4. However, these drugs are often accompanied by off-target problems or poor treatment outcomes, as well as systemic side effects and serious complications 5, 6. In recent years, DNA nanomedicine has received extensive attention in disease therapy. Antisense DNA, DNA aptamers, and specific DNA nanostructures have all demonstrated promising therapeutic effects and natural biocompatibility 7. As novel nanomaterials, DNA nanostructures possess programmable and easily modifiable properties, which provide significant advantages in nanomedicine design and combination therapies, positioning DNA-based therapies as a prospective treatment strategy for various diseases 8. However, several limitations of DNA restrict its therapeutic application, such as degradation in serum, rapid renal clearance and nonspecific hepatic aggregation 9. Therefore, there is an urgent need to develop a more stable and efficient therapy method for DNA nanostructures.

Current approaches to overcoming these challenges have focused mainly on the encapsulation of DNA in nanocarriers or the chemical modification of nucleotides. For example, lipid nanoparticles are commonly used in nucleic acid delivery to enhance stability, such as antisense oligonucleotides, plasmid DNA, etc. 10. Metal materials such as metal‒organic frameworks or nanozymes have been employed to improve the delivery efficiency of DNA nanomedicines 11. In addition, methods to increase targeting, such as the use of antibody oligonucleotide conjugates, effectively reduce the nonspecific hepatic aggregation of DNA nanomedicine 12. However, introduced modifications or chemical components may cause additional immune responses and cytotoxicity. Therefore, developing a highly stable and specific DNA-only nanomedicine delivery system may be a feasible way to address these issues.

Tetrahedral DNA nanostructures (TDNs) represent nucleic acid nanomaterials with a tetrahedral spatial structure and have been reported to possess a strong anti-inflammatory capacity 13-18. The specific spatial structure endows TDNs with excellent biological properties, such as enhanced cellular uptake efficiency, superior tissue permeability, and good biosafety profiles; these unique biological activities make TDNs potential therapeutic agents for the treatment of IBD. DNA aptamers, known for their flexibility, strong tissue infiltration, and high specificity, are also important components of DNA-based therapies. Toll-like receptor 4 (TLR4) is an important membrane receptor protein of immune cells for lipopolysaccharide (LPS)-induced inflammation 19. The TLR4 aptamer (ApTLR4) has been reported to be a highly selective, functional aptamer that can inhibit LPS-induced activation of the NF-κB pathway both *in vitro* and *in vivo* 20. Additionally, upon IBD status, high concentrations of extracellular ATP aggregate in the inflammatory microenvironment, which provides qualitative and quantitative information about pericellular injury to immune cells 21. This characteristic of ATP makes it possible for its DNA aptamers to be response elements for precise localization and response of the inflammatory sites. In addition to their anti-inflammatory and targeted effects, overcoming the nuclease environment *in vivo* presents another challenge for DNA nanomedicine. However, DNA hydrogels, which are characterized by high stability, precise self-assembly, programmability, and excellent biocompatibility, offer promising solutions for the delivery of pure DNA nanomedicine 22, 23. Moreover, oral administration is a preferable method of drug delivery in IBD patients because of its convenience, direct effect on the local mucosa, and ability to increase the local drug concentration. This approach can effectively prevent systemic drug exposure and nonspecific organ aggregation 24. Thus, considering all the above descriptions of DNA nanomedicines and the specific environment of IBD, it is reasonable to believe that a tailored DNA element integration platform may leverage their advantages to achieve a specific and highly efficient therapeutic effect in IBD.

**Figure 1:**
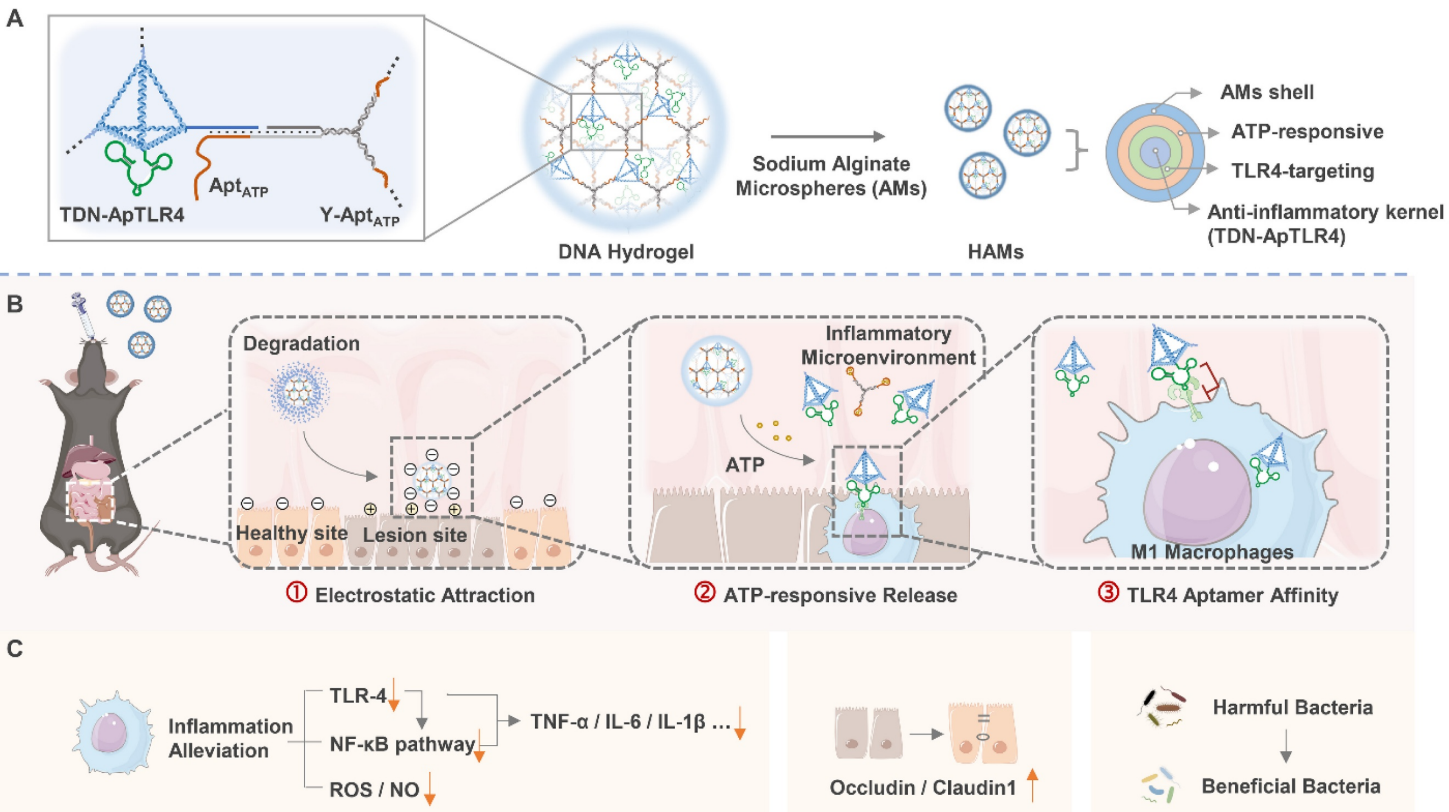
** Schematic diagram of programmed activated HAMs targeted therapy for IBD.** (A) Constitution and preparation of sodium alginate microcapsules (AMs) shelled with DNA hydrogels (HAMs). (B-C) Programmed activated HAMs and their anti-inflammatory effects. After oral administration, AMs provide a protective barrier, ensuring that HAMs withstand the harsh gastric milieu and exhibit controlled intestinal release. The released negatively charged DNA hydrogel is enriched at the inflammatory site by electronic attraction. Next, the high concentration of ATP in the inflammatory microenvironment initiates the release of TDN-ApTLR4 from the DNA hydrogel. Finally, ApTLR4 induces TDN targeting inflammatory immune cells to alleviate inflammation. HAMs achieve favorable therapeutic efficacy by decreasing the production of inflammatory factors (TNF-α, IL-6, and IL-1β), restoring the intestinal barrier, and modulating the gut microbiota.

In this study, we combined anti-inflammatory TDNs, ATP-responsive nano-scaffolds, and TLR4 pathway inhibitory aptamers to develop a drug-free HAMs system for the oral administration of targeted therapy for IBD. This system functions not only as a stable delivery vehicle for DNA therapeutics but also as a drug release platform that programmatically responds to the *in vivo* microenvironment. Unlike conventional approaches, where chemical hydrogels are used to load DNA or where DNA hydrogels are used to deliver chemical drugs, our system is composed entirely of DNA, which highly minimizes its immunogenicity. This DNA hydrogel system is assembled through DNA self-assembly, with aptamer-TDNs acting as nodes and ATP-responsive Y-shaped DNA as the scaffold. The network hydrogel structure effectively improved the stability of the functional DNA components. In addition, the entire DNA hydrogel is encapsulated in sodium alginate microcapsules (AMs) to form HAMs, which shield it from the harsh acidic environment of the stomach. Upon reaching the neutral environment of the intestine, AMs dissolve to release the DNA hydrogel. The released negatively charged DNA hydrogel is subsequently attracted to the positively charged inflamed intestinal regions. Next, the DNA hydrogel scaffold is disassembled by high concentrations of ATP in the inflammatory environment, releasing aptamer-TDNs, which are specifically designed to inhibit inflammation, along with a TLR4 aptamer that targets inflammatory immune cells, thereby increasing anti-inflammatory efficiency. HAMs demonstrate favorable therapeutic efficacy by decreasing the production of inflammatory factors (TNF-α, IL-6, and IL-1β), restoring the intestinal barrier, and modulating the gut microbiota in an IBD mouse model (Figure 1). Notably, the structure of the hydrogel improves the stability of DNA, and the step-by-step response design enables specific aggregation and sustained release of drugs at the disease location. Through this approach, we developed an intelligent, programmable DNA nanostructure for IBD treatment, achieving a strategy of DNA self-assembly, self-protection, and self-transport, thus enabling the construction and delivery of a biocompatible and efficient pure DNA nano-therapy system.

## Methods

### Materials

DMEM was purchased from Gibco. Cell culture flasks were purchased from NEST Biotechnology. LPS were obtained from Sangon Biotech. Dextran sodium sulfate (MW: 36,000-50,000 Da) was purchased from MP Biomedicals, Inc. Cell cryopreservation solution was obtained from Biochannel Biological Technology. Female mice were purchased from the Laboratory Animal Center of Shandong First Medical University (Jinan, China). All cells were obtained from the Cell Bank/Stem Cell Bank, Chinese Academy of Sciences. All animal experiments were approved by the Ethics Committee of Shandong First Medical University. All oligonucleotides were synthesized by Sangon Biotechnology Co., Ltd. or Genecfps (China).

### Synthesis of the DNA hydrogel

The DNA hydrogel was formed by hybridization of the Y scaffold with the sticky end on the TDN. Among them, TDNs were formed via a modified protocol 25. Specifically, tetrahedrons 1-4 were separately dissolved in TE buffer at a concentration of 100 μM. To form the tetrahedral DNA nanostructure, we mixed each strand equally in TM buffer, heated it to 95°C for 10 min and room temperature for 20 min, and then cooled it to 4°C for 20 min. The Y scaffold was constructed using the same protocol. Then, TDN was mixed with the Y scaffold at a ratio of 1:1 and incubated at 37°C for 24 h.

### Gel electrophoresis

The synthesis of the DNA hydrogel was verified by 3% agarose. 8 µL samples were prepared by mixing 5 µL of DNA hydrogel, 1 µL of Gel Red and 2 µL of loading buffer. Scanning was performed after 30 min of electrophoresis at 120 V via a Chemi Scope 6200 fluorescence chemiluminescence gel imaging system (Shanghai Qinxiang Scientific Instrument Co., Ltd.).

### Cell culture

RAW264.7 cells, Caco2 cells and HUVECs were cultured in DMEM supplemented with 10% fetal bovine serum. The cells were cultured at 37°C in a humidified incubator with 5% CO_2_.

### Flow cytometry

After being incubated with FAM-labeled TDN, ApTLR4, TDN-ApTLR4 or DNA hydrogel for 4 h, the RAW264.7 cells were washed with PBS and collected via centrifugation, and endocytosis was detected via flow cytometry (NovoCyte-A310, Agilent Biosciences Co., Ltd.).

### Confocal imaging

RAW264.7 cells were cultured in DMEM containing 10% FBS (VivaCell, Shanghai) and penicillin/streptomycin (1%) for 24 h and then added to FAM-TDN, FAM-ApTLR4, FAM-TDN-ApTLR4, the FAM-DNA Hydrogel or the DNA hydrogel at 37°C for 4 h. Next, the cells were stained with Hoechst, washed with PBS and fixed with paraformaldehyde solution. Finally, confocal laser scanning microscopy (Zeiss Celldiscoverer 7, Germany) was used for imaging.

### MTT

The cells were seeded in 96-well plates (1.0 × 10^4^ cells/well). The next day, the cells were treated with various concentrations of DNA hydrogel for 24 h. Afterward, the cells were washed with PBS, 10 µL of MTT (5 mg/mL) (KeyGEN BioTECH) was added for 4 h, and the mixture was dissolved in 100 µL of DMSO. The absorbance was measured via a microplate reader at 490 nm.

### ELISA

To assess the changes in TNF-α secreted by cells, RAW264.7 cells were treated with TDN, ApTLR4, TDN-ApTLR4, or DNA hydrogel for 4 h and then stimulated with LPS for 8 h, after which the cell supernatant medium was collected. The TNF-α levels were subsequently determined via a mouse ELISA kit (Shanghai Enzyme Linked Biotechnology Co., Ltd., China) following the manufacturer's guidelines.

### Real-time quantitative PCR

Total RNA was extracted via an RNA isolation kit (Keyoubo, Suzhou, China) according to the manufacturer's guidelines. cDNA was synthesized via a cDNA reverse transcription kit (Vazyme, Nanjing, China). Real-time quantitative PCR was subsequently performed with ChamQ SYBR Color qPCR Master Mix (Vazyme, Nanjing, China) in a Roche LightCycler 480 Real-Time PCR System (Roche, Switzerland). ACTB was used as an internal control gene and analyzed according to the 2^-∆∆Ct^ method. The primers used are shown in Table S2.

### Western blot analysis

For colon tissue samples, 3 × 10^5^ cells were harvested and homogenized in lysis buffer with protease and phosphatase inhibitor cocktails (TargetMol, USA). Protein quantification was performed using the BCA protein assay kit (Smart-Lifesciences). Then, the protein samples were desaturated at 100°C for 10 min with SDS loading buffer (Genscript Biotech, Nanjing). Then resolved via SDS‒PAGE and transferred to PVDF membranes, followed by 1 h of blocking with 5% skim milk. The membranes were incubated with primary antibodies at 4°C overnight. The next day, the sections were incubated with secondary antibodies for 1-2 h at room temperature. Finally, the bands were visualized via an enhanced chemiluminescence (ECL) reagent. Primary antibodies against TNF-ɑ (HuaBio), iNOS (HuaBio), Nrf2 (Abcam), p-IκBα (HuaBio), p-p65 (Bioworld), and ACTB (ABclonal) were used.

### Transwell migration assay

Caco2 cells were seeded on the apical side of the transwell plate and covered with 400 μL of basic medium (1×10^4^ cells/insert), and the basolateral side was covered with 600 μL of complete medium (10% FBS). TDN, ApTLR4, TDN-ApTLR4, and DNA hydrogel were added to both the apical and basolateral sides and incubated for 24 h. Then, the bottoms of the cell chambers were fixed with 4% paraformaldehyde for 30 min, stained with 0.1% crystal violet for 30 min, and imaged.

### *In vitro* wound healing assay

Caco2 cells were seeded in 12-well plates. When the cell confluence reached approximately 80%, a scratch wound was made with a sterile pipet tip, and the samples were then treated with TDN, ApTLR4, TDN-ApTLR4, or DNA hydrogel in basic medium for 48 h. The wound-healing percentages were measured via ImageJ.

### Preparation of alginate microspheres

TDN, ApTLR4, TDN-ApTLR4 and DNA hydrogel were mixed with a 2% alginate EDTA-Ca^2+^ solution. The alginate mixture was used as the aqueous phase, and the drop generation oil (FluidicLab, Shanghai) was used as the oil phase. The aqueous phase was dropped into the drop generation oil containing 1% acetic acid via the microfluidic platform. After being fixed for 20 min, the microspheres were isolated with demulsifiers (FluidicLab, Shanghai) and resuspended in PBS.

### DSS-induced model of colitis

Six-week-old female C57BL/6 mice were housed for one week before inclusion in the study. All the mice were randomly distributed into different groups. The mice received 3.5% (w/v) dextran sodium sulfate (DSS) supplemented in the drinking water for 5 days, followed by the addition of normal water. Healthy control mice were provided with normal water only. AMs, TDN@AMs, ApTLR4@AMs, DAMs and HAMs containing 0.3 nmol of DNA were then orally administered to the mice every two days. Body weight, stool consistency, and fecal occult blood were measured daily. On the last day of treatment, the mice were sacrificed, and the main organs, colon tissues and fecal samples were collected. The main organs were fixed for histological assessment. Colon length was measured, and the samples were gently washed with physiological saline. Two pieces (0.5 cm in length) of the distal sections were subsequently fixed or quickly frozen in liquid nitrogen for histological assessment, immunofluorescence staining and DHE analysis. Fecal samples were sent to Majorbio (Shanghai, China) for microbiome analysis.

### Fluorescence imaging and biodistribution

The mice with colitis were fasted for 24 h, and their abdominal hair was shaved before imaging. The *in vivo* biodistributions of Cy5-labeled DAMs and HAMs at the intestinal site were imaged via an *in vivo* imaging system (Tanon ABL-X6, Shanghai). For *ex vivo* imaging, the mice were euthanized after oral administration of the FAM-labeled DAMs or HAMs. The colon was subsequently collected and imaged with an *in vivo* imaging system (Tanon ABL-X6, Shanghai).

### Histopathology

Colon tissue samples and major organs, including the heart, liver, spleen, lungs, and kidney, were fixed in 4% paraformaldehyde and embedded in paraffin. Tissue sections with a thickness of 4 µm were stained with Hematoxylin and eosin (H&E) by hematoxylin and eosin staining kit (APExBIO Technology LLC) and observed under a light microscope.

For the immunofluorescence analysis, the colon sections were subjected to antigen retrieval and blocking; incubated with primary antibodies against TNF-ɑ (HuaBio), Occludin (HuaBio), Claudin1 (HuaBio), iNOS (HuaBio), p-p65 (Bioworld), and Nrf2 (ABclonal); and then incubated with secondary antibodies. The slides were subsequently washed three times with PBS, stained with DAPI solution and analyzed via wide-field fluorescence microscopy.

### Long-term toxicity test

Six-week-old C57BL/6 mice were randomly distributed into control and HAMs treatment groups (50% female and 50% male). The HAMs in the treatment group were orally administered HAMs containing 3 nmol of DNA every other day for 5 weeks. Body weight and food intake were recorded throughout the test. After the final treatment, the mice were sacrificed. The main organs were harvested for H&E staining. Blood was collected for routine clinical analysis.

### RNA sequencing

Total RNA from mouse colon tissue was isolated via TRIzol (Invitrogen, Thermo Fisher Scientific). Library construction and sequencing were performed by Majorbio (Shanghai, China). After the transcriptome library was constructed, sequencing was performed via the Illumina HiSeq X Ten/NovaSeq 6000 sequencer. The differential expression and functional enrichment analyses were subsequently performed via the Majorbio biological cloud platform (https://cloud.majorbio.com/page/tools/).

### Microbiome analysis

Fecal samples were collected and immediately frozen in liquid nitrogen for subsequent sequencing analysis of the intestinal microbiome. Library construction and sequencing were performed by Majorbio (Shanghai, China). The bacterial 16S rRNA gene (V3-V4 region) was amplified via the primer pairs 338F (5′- ACTCCTACGGGAGGCAGCAG- 3′) and 806R (5′- GGACTACHVGGGTWTCTAAT- 3′). Data analysis was performed via the Majorbio Biological Cloud platform (https://cloud.majorbio.com/page/tools.html).

### Statistical analysis

All experiments were independently replicated at least three times. All the experimental data were statistically analyzed with GraphPad Prism software version 9.0 (San Diego, USA). Student's t-test or one-way analysis of variance (ANOVA) followed by Tukey post hoc analysis was used to determine the significant differences between the different groups. The results are presented as the means ± standard deviations (SD). *p < 0.05, **p < 0.01, ***p < 0.001, NS, not significant.

## Results

### Design and characterization of the anti-inflammatory DNA hydrogel

**Figure 2:**
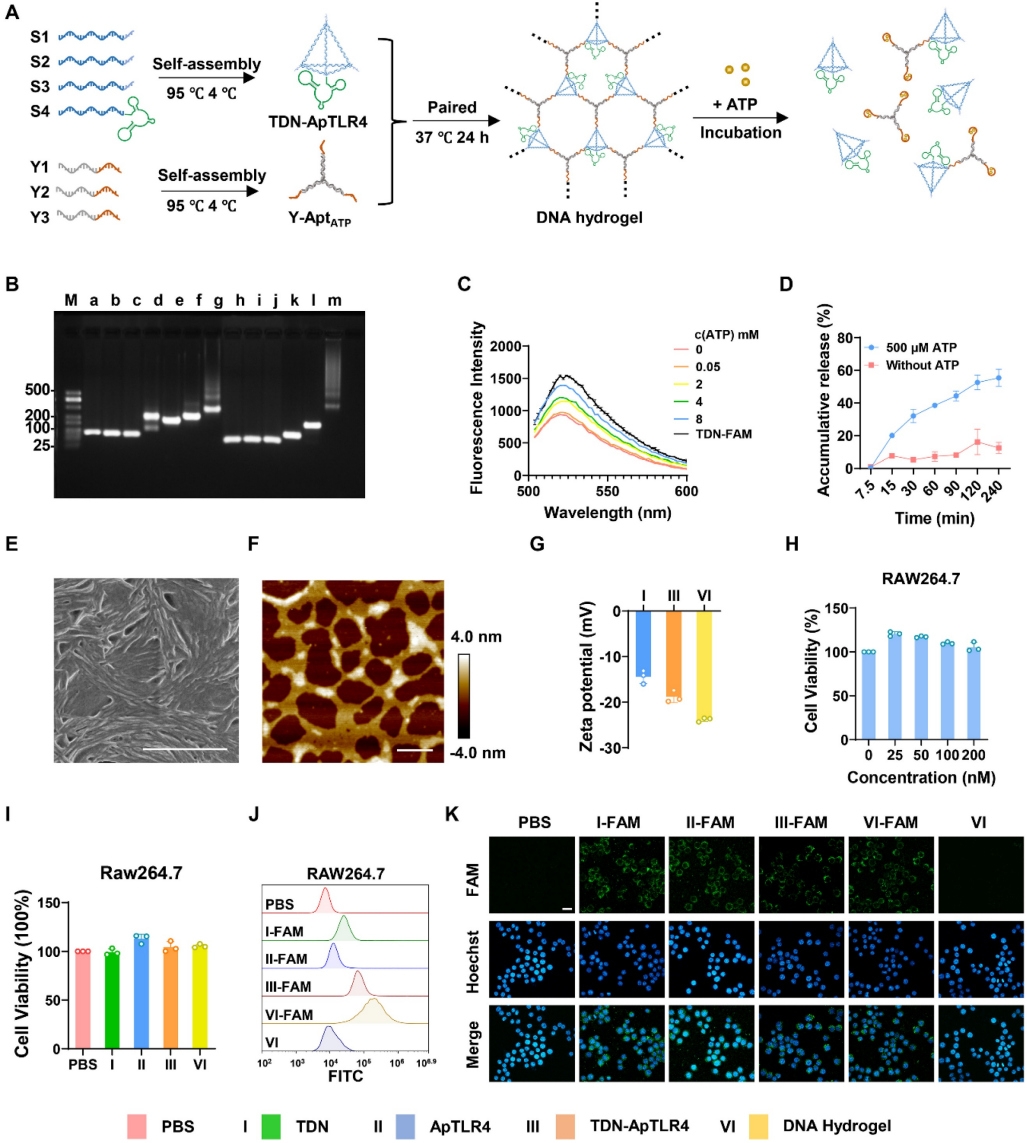
** Characterization of the DNA hydrogel.** (A) Schematic of the synthesis process of the DNA hydrogel and ATP-responsive release. (B) Agarose gel electrophoresis of the synthesized DNA hydrogel (a: S1, b: S2, c: S3, d: S4, e: S1+S2, f: S1+S2+S3, g: S1+S2+S3+S4 (TDN- ApTLR4), h: Y1, i: Y2, j: Y3, k: Y1+Y2, l: Y1+Y2+Y3 (Y-AptATP scaffold), m: TDN-AptTLR4+ Y-AptATP scaffold (DNA Hydrogel). (C) Fluorescence intensity of the DNA hydrogel incubated with various concentrations of ATP. (D) Release rate of the DNA hydrogel in response to ATP. (E) SEM image of the DNA hydrogel; scale bar, 1.00 μm. (F) AFM image of the DNA hydrogel; scale bar, 300 nm. (G) Zeta potentials of TDNs, TDN-ApTLR4, and the DNA hydrogel. (H) Biocompatibility of various concentrations of DNA hydrogel toward RAW264.7 cells. (I) Biocompatibility of different nanostructures toward RAW264.7 cells. (J) Cellular uptake efficiency of different nanostructures by RAW264.7 cells. (K) Fluorescence images of the cellular uptake of different nanostructures by RAW264.7 cells (scale bar: 20 μm).

Here, DNA hydrogels were constructed through a careful annealing process via self-assembly of TDN-ApTLR4 and Y-AptATP scaffolds. As shown in Figure 2A, TDN-ApTLR4 self-assembled from four designed single-stranded DNAs (ssDNAs) via base pairing, and ApTLR4 was added to the 3′ ends of S4. The Y-AptATP scaffold was synthesized from three ssDNAs with an active AptATP chain. TDN-ApTLR4 was circularly hybridized with the three vertices of the Y-AptATP scaffold to form the DNA hydrogel. In this hydrogel, ApTLR4 was chosen because of its high selectivity for immune cells and its inhibitory role in TLR4 activation during LPS-induced inflammation 26. Given that ATP was highly aggregated at inflammation sites, it disintegrated the DNA hydrogel to release TDN-ApTLR4 by competing with the aptamer on the hydrogel Y scaffold.

To verify the correct formation of the DNA hydrogel, gel electrophoresis was first performed. As shown in Figure 2B, lane m exhibited a supershift and diffuse tailing, indicating successful assembly of the DNA hydrogel. In addition, to ensure the high efficiency of DNA hydrogel disintegration in response to ATP, we optimized the number of complementary sticky terminal bases of the TDN-ApTLR4 and Y-AptATP scaffolds. As shown in Figure S1, more than 88% of the hybrid strands of S1-2 and Y1-2 were unraveled, suggesting that complementary base number 13 is appropriate for subsequent experiments. When the ratio of TDN-ApTLR4 to Y-AptATP scaffold was 1:1 and the mixture was incubated for 24 h, the DNA hydrogel had the highest synthesis efficiency, with no free TDN-ApTLR4 or Y-AptATP scaffold remaining. (Figure S2A-B). Furthermore, an ATP-responsive fluorescence-releasing DNA hydrogel was synthesized by labeling quenching groups on Y-AptATP scaffolds and fluorescent groups on ATP aptamers. The ATP-responsive disintegration of the DNA hydrogel was then verified by gradient analysis of the ATP concentration (Figure 2C and Figure S3A). The results revealed that the release rate of TDN-ApTLR4 reached approximately 60% with 500 μM ATP in 240 min in a neutral environment (pH 7.5) (Figure 2D). In addition, to investigate whether pH influences the release rate of TDN-ApTLR4, we conducted experiments under low-pH conditions. As shown in Figure S3B, the release rate of TDN-ApTLR4 at pH 6.5 was comparable to that at pH 7.5. This finding indicated that pH had no influence on the ATP-controlled release of TDN-ApTLR4. Scanning electron microscopy (SEM), atomic force microscopy (AFM), and transmission electron microscopy (TEM) images intuitively revealed the network structure of the DNA hydrogel (Figure 2E-F, Figure S4). In addition, the individual structures of the TDN-ApTLR4 and Y-AptATP scaffolds were characterized via AFM (Figure S5). The zeta potentials of TDN, TDN-ApTLR4, and the DNA hydrogel were all less than -10 mV, whereas the DNA hydrogel had the lowest potential of -23.8 ± 0.19 mV; the nature of the negative potential was more attractive for the positively charged enteritis microenvironment (Figure 2G).

Biocompatibility is a necessary property of nanodrugs. As shown in Figure 2H and Figure S6A-B, the DNA hydrogel had no significant effect on the growth of Caco2 cells (human rectal cancer cell line), RAW264.7 cells (mouse mononuclear macrophages), or HUVECs (human umbilical vein endothelial cells). In addition, 200 nM TDN, ApTLR4, TDN-ApTLR4, or DNA hydrogel was applied to RAW264.7 cells with or without LPS stimulation, and cell viability was not affected (Figure 2I and Figure S6C). Moreover, cell flow cytometry and confocal laser scanning microscopy revealed that FAM-labeled TDN, ApTLR4, TDN-ApTLR4, and the DNA hydrogel produced intense FAM signals in RAW264.7 cells (Figure 2J-K), indicating that all four nanostructures had good cellular uptake efficiency.

### Anti-inflammatory capacities of anti-inflammatory DNA hydrogels *in vitro*

**Figure 3:**
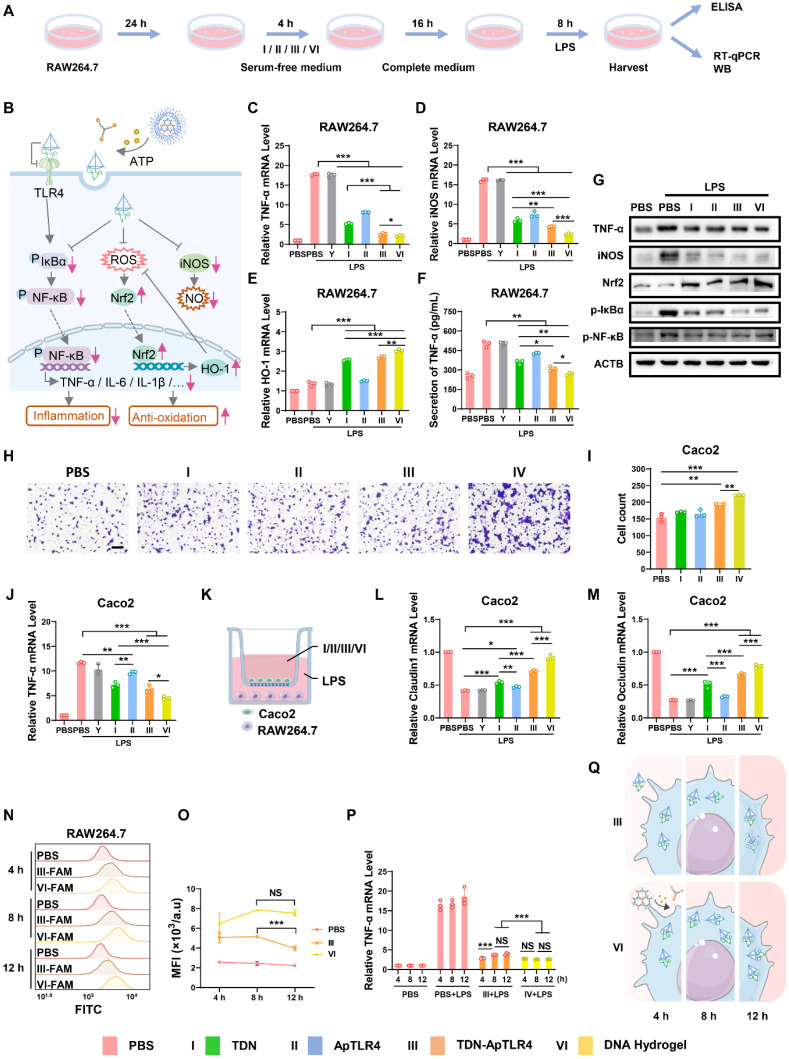
** Anti-inflammatory effects of the DNA hydrogel.** (A) Diagram of the experimental procedures. (B) Schematic diagram of key inflammatory signaling regulators involved in the anti-inflammatory effect of the DNA hydrogel. mRNA expression of TNF-α (C), iNOS (D), and HO-1 (E) in RAW264.7 cells after different treatments. (F) Detection of TNF-α in the supernatants of RAW264.7 cells after different treatments. (G) Western blotting was used to analyze the protein expression of related inflammatory regulators in RAW264.7 cells after different treatments. (H-I) Transwell showed the migration of Caco2 cells under different treatments. (J) mRNA expression of TNF-α in Caco2 cells after different treatments. (K) Scheme of the RAW264.7/Caco2 coculture model. (L-M) mRNA expression of Claudin-1 and Occludin in Caco2 cells in the coculture model after different treatments. (N‒O) Flow cytometry was used to monitor TDN-ApTLR4 / DNA hydrogel uptake in RAW264.7 cells after different treatment times, and the results were statistically analyzed. (P) mRNA expression of TNF-α in RAW264.7 cells after different treatments for different durations. (Q) Schematic diagram of TDN-ApTLR4 and the DNA hydrogel in RAW264.7 cells after increasing treatment time. Y, Y-AptATP scaffold.

To evaluate the therapeutic potential of the DNA hydrogel, we first assessed its anti-inflammatory role at the cellular level. RAW264.7 cells were used as a model for significant inflammatory responses after LPS stimulation. The cells or media were harvested after treatment with or without the Y-AptATP scaffold, TDN, ApTLR4, TDN-ApTLR4, or DNA hydrogel following LPS challenge, and inflammation-related factors were measured (Figure 3A-B). As shown in Figure 3C and Figure S7, the levels of the inflammatory factors TNF-α, IL-6, and IL-1β were significantly increased in RAW 264.7 cells after LPS challenge, whereas they were sharply decreased in TDN and TDN-ApTLR4, especially in the DNA hydrogel-treated group, and the Y-AptATP scaffold had no individual effects. Previous studies have shown that TDNs possess anti-inflammatory and reactive oxygen species (ROS) and nitric oxide (NO) scavenging capabilities 13-18. Thus, inducible nitric oxide synthase (iNOS) (the main mediator of nitric oxide production) mRNA expression was detected in these groups, whereas heme oxygenase-1 (HO-1) (an important regulator of the ROS level) exhibited the opposite trend (Figure 3D-E). Similarly, the levels of cytokines of TNF-α in the supernatants in DNA hydrogel treated cells were also significantly decreased (Figure 3F). Moreover, western blot analysis further confirmed the protein levels of related proteins. As shown in Figure 3G, the expression patterns of TNF-α, iNOS, and Nrf2 (upstream regulators of HO-1) showed similar expression patterns to mRNA level, as well as p-IκBα and p-p65. Taken together, these results indicated that DNA hydrogel treatment induced an obvious anti-inflammatory effect by inhibiting the NF-κB pathway, iNOS level, and Nrf2/HO-1 signaling.

After exploring the anti-inflammatory effects of the DNA hydrogel in RAW264.7 macrophages, we next evaluated its anti-inflammatory effects in Caco2 cells, a commonly used model for colonic epithelial tissue. According to the results of the cell migration and scratch assays shown in Figure 3H-I and Figure S8, TDN, TDN-ApTLR4, and DNA hydrogel treatment significantly promoted the migration of Caco2 cells. Notably, the DNA hydrogel treatment group presented the highest migratory capacity among the groups. These results suggested that the DNA hydrogel promoted the migration of intestinal epithelial cells (IECs), potentially accelerating the reepithelization of damaged intestinal mucosa and thus facilitating ulcer healing. To further investigate the anti-inflammatory effects of the DNA hydrogel in Caco2 cells, an LPS-induced inflammation model was established, and the Caco2 cells were treated with those DNA materials. As shown in Figure 2J and Figure S9, the mRNA levels of TNF-α, IL-6 and IL-1β were significantly decreased after treatment with TDN, TDN-ApTLR4, or DNA hydrogel. Consistent with the results in RAW264.7 cells, the DNA hydrogel treatment group presented the lowest expression levels among all the groups, indicating its superior anti-inflammatory efficacy. In addition, to simulate the inflammatory pathological microenvironment of IBD, a RAW264.7 cell and Caco2 cell coculture model was established (Figure 3K). After induction with LPS and treatment with those DNA materials, the expression of two important tight junction proteins, Occludin and Claudin1, which are closely related to the integrity of the intestinal barrier, was measured. The data revealed that the DNA hydrogel treatment significantly increased the levels of the Occludin and Claudin1 mRNAs (Figure 3L-M). These results indicated that the DNA hydrogel accelerated epithelial wound healing and facilitated the reconstruction of mucosa in IBD patients.

Furthermore, considering the different stabilities of the DNA hydrogel and TDN, we speculated that the DNA hydrogel had advantages in terms of its anti-inflammatory effects for long-term therapy. Therefore, we extended the coincubation time of the TDN-ApTLR4 / DNA hydrogel with cells and subsequently examined the fluorescence stability and the mRNA levels of the relevant key regulators (Figure S10A). Flow cytometry revealed that after 12 h of treatment, DNA hydrogel treated cells presented stronger and more persistent fluorescence, whereas TDN-treated cells presented a decrease in fluorescence intensity, suggesting that the DNA hydrogel can be more easily taken up by cells and is more stable in cells (Figure 3N-O). In addition, DNA hydrogel-treated cells presented more durable and stable inflammation inhibition (Figure 3P-Q, Figure S10B-C).

Taken together, our results showed that the DNA hydrogel had an efficient anti-inflammatory effect on macrophages and IECs, suggesting its potential therapeutic efficacy in IBD treatment.

### Preparation and characteristics of the sodium alginate microcapsule-coated DNA hydrogel

**Figure 4:**
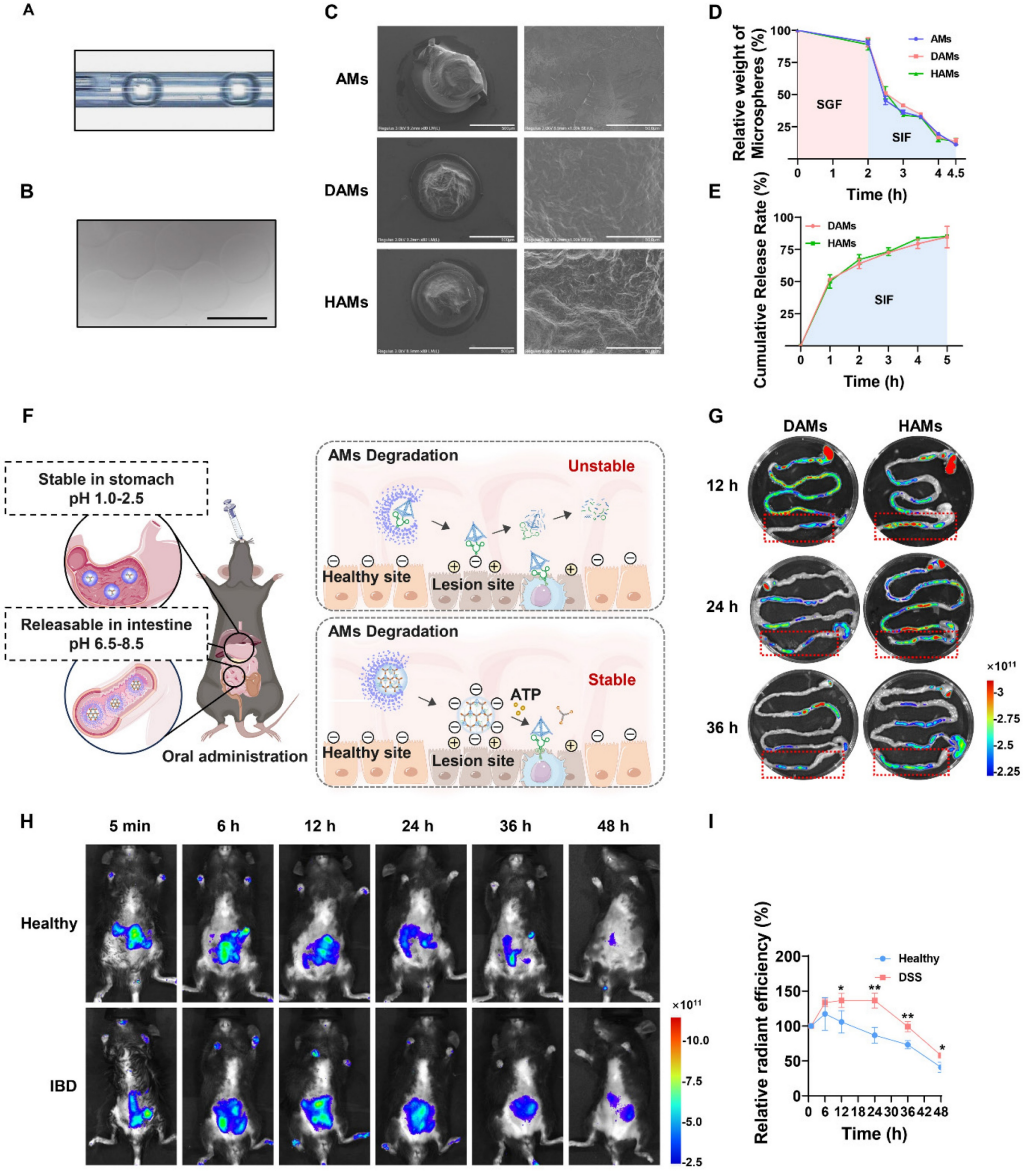
** Preparation and characteristics of sodium alginate microcapsule-coated DNA hydrogels.** (A) Real-time images of the droplet formation procedure in the microfluidic device. (B) Optical image of the microparticles. Scale bar: 500 μm. (C) SEM image of AMs/DAMs/HAMs. DAMs, AMs coated with TDN-ApTLR4; HAMs, AMs coated with DNA hydrogel. Scale bar: left, 500 μm; right, 50 μm. (D) Relative weights of AMs/DAMs/HAMs after incubation with SGF and SIF. SGF, simulated gastric fluid; SIF, simulated intestinal fluid. (E) Cumulative release rate of DNA from DAMs and HAMs. (F) Hypothetical schematic of IBD mice after the oral administration of DAMs or HAMs. (G) Fluorescence images of the mouse gastrointestinal tract after the oral administration of doses of DAMs and HAMs. (H-I) Fluorescence images and relative radiant efficiency of healthy and IBD mice at different time after oral administration of HAMs.

In the above part, we successfully constructed an anti-inflammatory DNA hydrogel with TDN-ApTLR4 to improve stability and verified that the DNA hydrogel had an obvious anti-inflammatory effect *in vitro*. Next, we investigated the distribution and inflammation site-targeting effects of the DNA hydrogel compared with those of TDN-ApTLR4. Oral anti-inflammatory drugs are the preferred and widely accepted method for IBD patients because of their convenience, safety, and direct delivery of therapeutic agents to the colonic mucosa 27. The key challenge in the oral administration of DNA nanomedicines is to improve their ability to resist the digestion of complex gastrointestinal fluids during drug delivery. AMs are widely used as oral drug delivery systems for controlling drug release in the intestine. When exposed to gastric acid, AMs are cross-linked with Ca^2+^ to form an insoluble gel. After reaching the neutral and low-ion concentration intestinal environment, the molecular chains of AMs gradually expand and dissolve, thereby releasing the loaded drugs 28, 29. In addition to their good biocompatibility and size controllability, AMs not only possess a high drug loading capacity but also benefit from mild preparation conditions, which effectively helps maintain the original structure of the encapsulated substance 30. Here, TDN-ApTLR4 and the TDN-ApTLR4 hydrogel were loaded into sodium alginate-assisted AMs to form DNA nanostructure composite microspheres generated via the microfluidics method. As shown in Figure S11A, EDTA-Ca^2+^ was mixed into the sodium alginate solution, and after being cleaved by the oil phase, the microdroplets were internally crosslinked by the acidic oil phase, which induced Ca^2+^ release and the formation of AMs. Figure 4A shows the formation of microdroplets cleaved by the oil phase, and the droplets then rapidly formed gelled AMs (Figure 4B). The particle size of AMs was suitable for mouse oral administration (~420 μm) (Figure S11B). Next, TDN-ApTLR4 and the DNA hydrogel were mixed in sodium alginate solution to generate AM-coated TDN-ApTLR4 and the DNA hydrogel (denote DAMs and HAMs, respectively). The SEM images of the structures of the AMs, DAMs, and HAMs are shown in Figure 4C. To evaluate the status of AMs, DAMs, and HAMs in the environment of the GI tract, these microspheres were incubated with simulated gastric fluid (SGF) for 2 h and then further incubated with simulated intestinal fluid (SIF) for 2.5 h to simulate the process of food in the digestive tract. As shown in Figure 4D, the weights of AMs, DAMs, and HAMs had no distinct change in SGF and rapidly decreased in SIF. In addition, the concentration of DNA in the solution released from both the DAMs and HAMs rapidly increased during incubation with SIF, and the cumulative release rate reached more than 80% after 5 h of SIF incubation (Figure 4E). These results indicated that the AM-coated TDN-ApTLR4 / DNA hydrogel protected the DNA nanostructure from premature release in the gastric environment and achieved targeted release in the intestinal environment.

To verify that the AMs-coated DNA hydrogel resisted digestive fluid *in vivo* and thereby targeted the colitis site, DSS-treated IBD mice were orally administered Cy5-labeled DAMs/HAMs. The entire GI tract of each mouse was subsequently collected at the final point for *in vitro* imaging. As shown in Figure 4F-G, a large amount of fluorescence was observed in the intestine. Interestingly, the fluorescence intensity in the colon was significantly greater in the HAMs group than in the DAMs group, indicating that, compared with free TDN-ApTLR4, the AMs-coated DNA hydrogel had greater resistance to digestive fluids and better targeting of the colitis site. The stability of the DNA hydrogel was also validated in SIF (Figure S12). Furthermore, to observe the targeting of HAMs to inflammatory sites, healthy and DSS-treated IBD mice were orally administered HAMs. As shown in Figure 4H-I, the fluorescence intensity was significantly stronger in the IBD mice than in the healthy mice at 12 h after the oral administration of HAMs, and fluorescence was still observable in the DSS-treated mice after 48 h. These results suggested that the inflammation-targeting properties of HAMs prolong their residence time within the colon.

### Therapeutic efficacy of HAMs in IBD model mice

**Figure 5:**
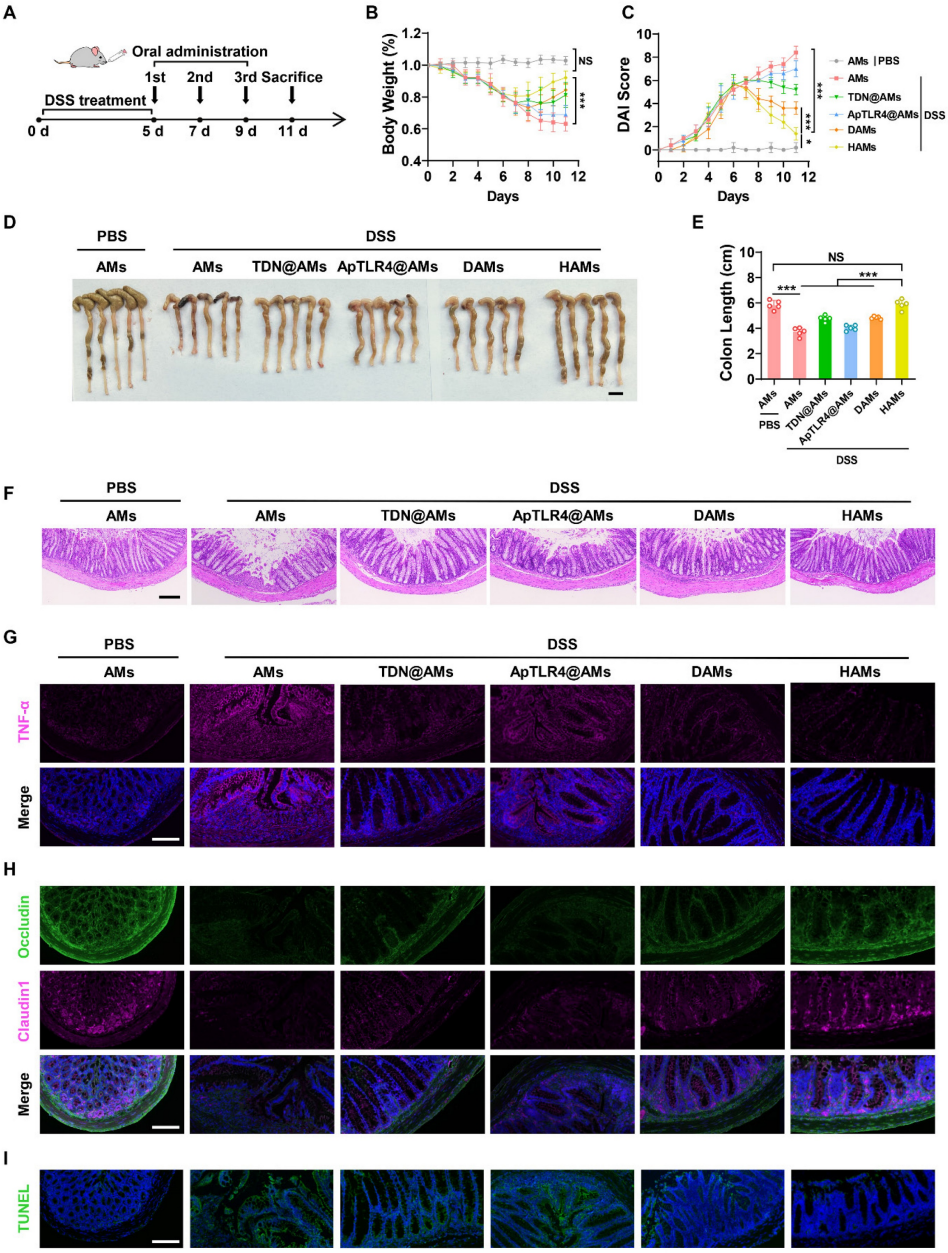
** Therapeutic efficacy of HAMs in IBD model mice.** (A) Schematic diagram outlining the experimental design. Percentage change in the body weights (B) and disease activity index (DAI) scores (C) of IBD and healthy control mice in response to different treatments. (D-E) Photographs of the colons of IBD or healthy control mice under different treatments and the corresponding statistical columns. Scale bar, 1 cm. (F) H&E staining of the colons of IBD or healthy control mice subjected to different treatments. Scale bar, 100 μm. (G) Immunofluorescence staining of TNF-α in colon sections from IBD or healthy control mice subjected to different treatments. Scale bar, 100 μm. (H) Immunofluorescence staining of two important tight junction proteins, Occludin and Claudin1, in the colons of IBD or healthy control mice subjected to different treatments. Scale bar, 100 μm. (I) Terminal deoxynucleotidyl transferase-mediated deoxyuridine triphosphate nick end labeling (TUNEL) staining of the colons of IBD or healthy control mice subjected to different treatments. Scale bar, 100 μm.

Encouraged by the impressive anti-inflammatory performance of the DNA hydrogel *in vitro*, as well as the precise intestinal delivery and inflammatory-targeting effects of its oral formulation (HAMs), we further investigated their therapeutic efficacy *in vivo*. Specifically, after oral administration of 3% DSS for 5 days, we orally administered AMs, AMs coated with TDN (TDN@AMs), AMs coated with ApTLR4 (ApTLR4@AMs), DAMs, or HAMs on days 5, 7, and 9, respectively (Figure 5A). The weight of each mouse was recorded daily, and the disease activity index (DAI) score was assessed to determine the severity of colitis. As shown in Figure 5B-C, HAMs inhibited body weight loss and downregulated the DAI score in IBD mice. Notably, the body weights of HAMs treated mice were similar to those of the healthy control mice on day 11 (Figure 5B). After the mice were sacrificed, the colons of the mice were collected, photographed, and measured. Alterations in colon length are pivotal indicators of the inflammatory response in IBD mouse models and reflect the extent of intestinal tissue damage and overall disease severity 31. The colon length in the HAMs group was significantly longer than that in the other treatment groups and was not significantly different from that in the healthy control group (Figure 5D-E). These results clearly demonstrated the strong preventive effect of HAMs on colitis.

Furthermore, pathological analysis of colon sections was performed to further verify the recovery of the inflammatory treatment. H&E staining revealed that HAMs treatment helped maintain the integrity of the colonic epithelium, reduced the infiltration of inflammatory cells, and significantly relieved symptoms of colonic inflammation (Figure 5F). Moreover, to elucidate the underlying therapeutic molecular mechanisms, we further examined the expression levels of inflammation-related markers and key molecules in colon sections.

Consistent with the *in vitro* results, colon sections showed a significant decrease in the level of TNF-α in the HAMs-treated group, indicating that inflammation in HAMs-treated IBD mice was apparently alleviated compared with that in DSS-treated mice (Figure 5G). In addition, the expression of iNOS and Nrf2 was also consistent with the results of the cell experiments (Figure S13A), and the ROS level was confirmed by dihydroethidium (DHE) staining (Figure S13B). These results highlighted the role of the DNA hydrogel in NF-κB signaling inhibition and NO and ROS scavenging in colon tissue. Interestingly, we also noted that the epithelium was relatively intact in HAMs treatment group compared with the DSS group. Thus, the expression of two important tight junction proteins, Occludin and Claudin1, was evaluated. As shown in Figure 5H, much greater expression of Occludin and Claudin1 was observed in the groups treated with HAMs. In addition, we further revealed the apoptosis of colon tissues via terminal deoxynucleotidyl transferase-mediated deoxyuridine triphosphate nick end labeling (TUNEL) staining. The results revealed that the green TUNEL fluorescence intensity greatly decreased in the HAMs treatment group, suggesting that apoptosis was notably reduced at the sites of colitis (Figure 5I). These findings suggested that HAMs could be involved in colon tissue repair. Moreover, after treatment with different microspheres, tissue morphology of major organs was noted as no observable changes, indicating its favorable biosafety profile (Figure S14). In summary, AMs coated DNA hydrogels (HAMs) offer efficacy therapeutic effects in IBD mice by inhibiting the release of inflammatory factors, scavenging ROS and NO, and facilitating colon tissue repair.

### Long-term toxicology evaluation of HAMs *in vivo*

Next, the safety and long-term toxicity of HAMs were investigated (Figure S15A). Body weight and food intake were recorded, which usually reflect the health condition of the treated mice. As shown in Figure S15B, the average body weights gradually increased during the treatment period and were not significantly different from those of the control groups. In addition, our data revealed that the amount of food intake did not significantly differ between HAMs-treated and PBS-treated mice (Figure S15C), suggesting good tolerance of the mice toward HAMs at the tenfold-fold therapeutically efficacious dose. Furthermore, blood parameters were tested to evaluate the influence of long-term HAMs treatment on mice (Figure S16). A complete blood sample analysis revealed that after 5 weeks of treatment, all parameters, such as white blood cells (WBCs), red blood cells (RBCs), and hemoglobin (HBG), were normal (Figure S16A-C). In addition, the hematocrit (HCT), mean corpuscular volume (MCV), mean corpuscular hemoglobin (MCH), and mean corpuscular hemoglobin concentration (MCHC) did not differ from those of the PBS control (Figure S16D-G). These data indicated that there was no significant risk associated with HAMs treatment. Furthermore, serum biochemical analysis revealed that all liver function markers (ALT, AST, and ALP) and kidney function markers (e.g., CREA) remained within the reference range, indicating no obvious injury to these organs (Figure S16H-I). Based on these results, tissue damage was further evaluated by H&E staining of the major organs after 5 weeks of treatment (Figure S17), and the results revealed no obvious histological damage to any organ. Collectively, these data suggest that there is no significant long-term toxicity of HAMs at the present dosage for up to 5 weeks.

### Transcriptome analysis reveals the underlying mechanism of HAMs treatment

**Figure 6:**
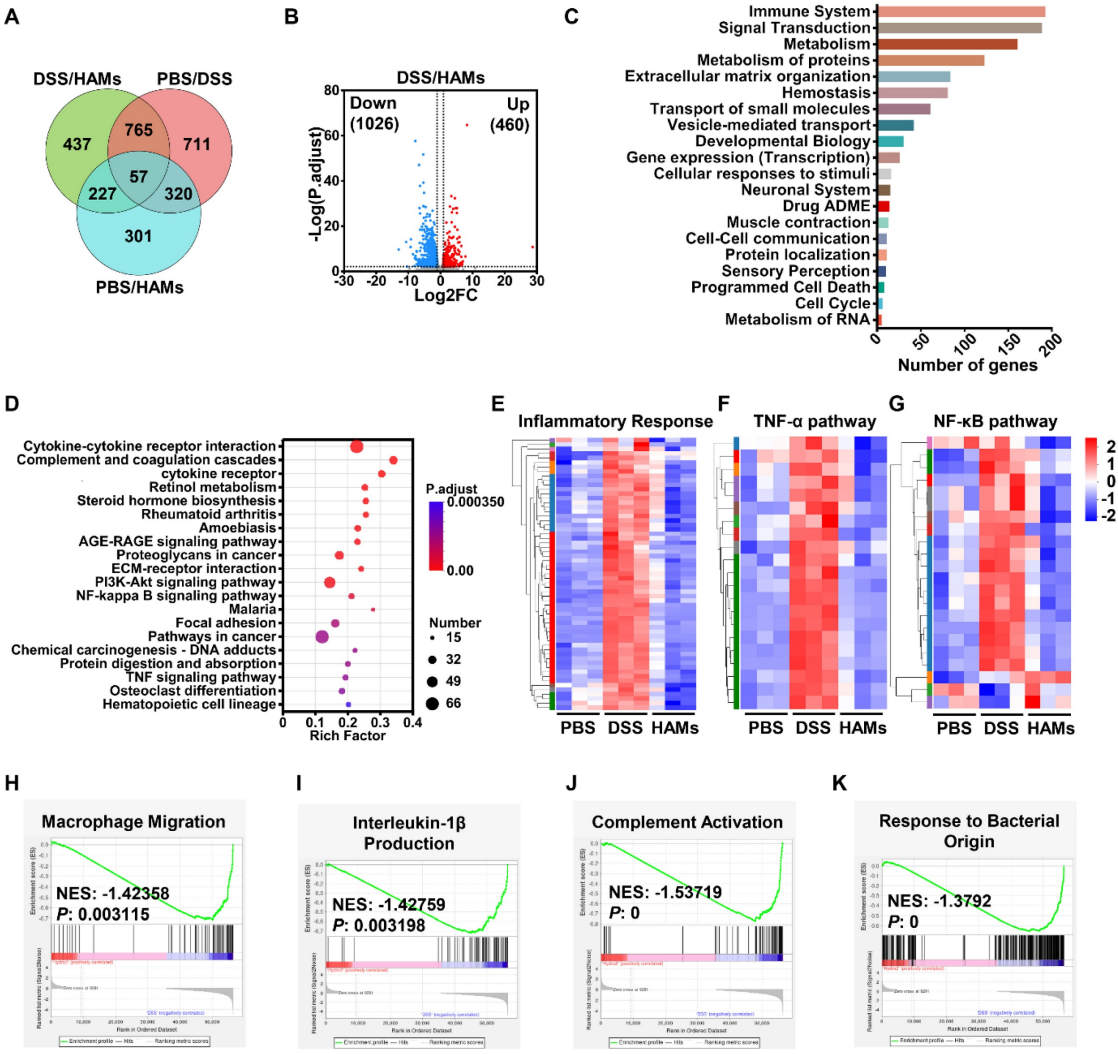
** mRNA sequencing of intestinal tissues from IBD mice after treatment with HAMs.** (A) Venn diagram showing the number of differentially expressed genes (DEGs) in pairs between PBS-treated (healthy mice), DSS-treated (IBD mice), and HAM-treated mice. (B) Volcano plot of DEGs between the DSS and HAMs groups. FC, fold change. (C) Reactome annotation analysis of DEGs in the DSS group versus the HAMs group. (D) KEGG pathway enrichment analysis of DEGs in the DSS group versus the HAMs group. Heatmap of markers for the inflammatory response pathway (E), TNF-α pathway (F), and NF-κB pathway (G) in the PBS, DSS, and HAMs groups. Enrichment plots from GSEA analyses of gene sets for “Macrophage Migration” (H), “Interleukin-1β Production” (I), “Complement Activation” (J), and “Response to Bacterial Origin” (K). NES, normalized enrichment score. |NES| > 1 and p value < 0.05 were considered statistically significant in the PBS group versus the HAMs group.

To dissect the molecular mechanisms of the therapeutic effect of HAMs on IBD, we performed transcriptome analysis of HAMs-treated IBD models. The differential expression gene analysis (p < 0.01, |FC| > 2.0) of the healthy (PBS), IBD (DSS), and HAMs treatment groups revealed a total of 1913 differentially expressed genes (DEGs) between the PBS and DSS groups, 1486 between the HAMs treatment and DSS groups, and 905 between the HAMs treatment and PBS groups (Figure 6A). Volcano plots highlighted the DEGs between the DSS and HAMs treatment groups, with 1026 downregulated genes and 460 upregulated genes (Figure 6B). Gene Ontology (GO) enrichment analysis revealed that the DEGs were enriched mainly in the immune system, signal transduction, and metabolism, as well as programmed cell death and the cell cycle, which are associated with IBD biological processes (Figure 6C). Moreover, Kyoto Encyclopedia of Genes and Genomes (KEGG) enrichment analysis revealed the pathways influenced by HAMs treatment, which were enriched in immune response pathways, including cytokine‒cytokine receptor interactions, complement and coagulation cascades and the TNF signaling pathway; IBD therapeutic target-related pathways, including the PI3K‒Akt signaling pathway and the NF‒κB signaling pathway; and ECM‒receptor interactions, which are important for promoting M1 macrophage polarization in IBD 32 (Figure 6D). To further characterize the therapeutic effect of HAMs, the expression profiles of specific pathway gene sets were analyzed. As shown in Figure 6E-G and Figure S18A, heatmaps revealed that the inflammatory response, TNF-α pathway, NF-κB signaling pathway, and immune response gene sets were obviously downregulated after treatment with HAMs, and the gene expression in the HAMs group reached a comparable level to that in the healthy control (PBS) group. Moreover, gene set enrichment analysis (GSEA) revealed that macrophage migration (Figure 6H), IL-1β production (Figure 6I), IL-6 production (Figure S18B), and complement activation were suppressed in the HAMs-treated group (Figure 6J), highlighting the role of HAMs-mediated inflammatory immune remodeling. In addition, consistent with the results of the pathway study in cells (Figure 3), HAMs also regulated nitric oxide and superoxide metabolic processes (Figure S18C-D), which may be the effect of TDN. The apoptotic signaling pathway (Figure S18E) was decreased in the HAMs treatment group, indicating that HAMs could inhibit IEC apoptosis. Furthermore, GSEA revealed that “response to bacterial origin” signaling was repressed in the HAMs-treated group (Figure 6K), suggesting that HAMs may influence the gut microbiota in IBD patients.

### Regulation of the gut microbiome by HAMs treatment

**Figure 7:**
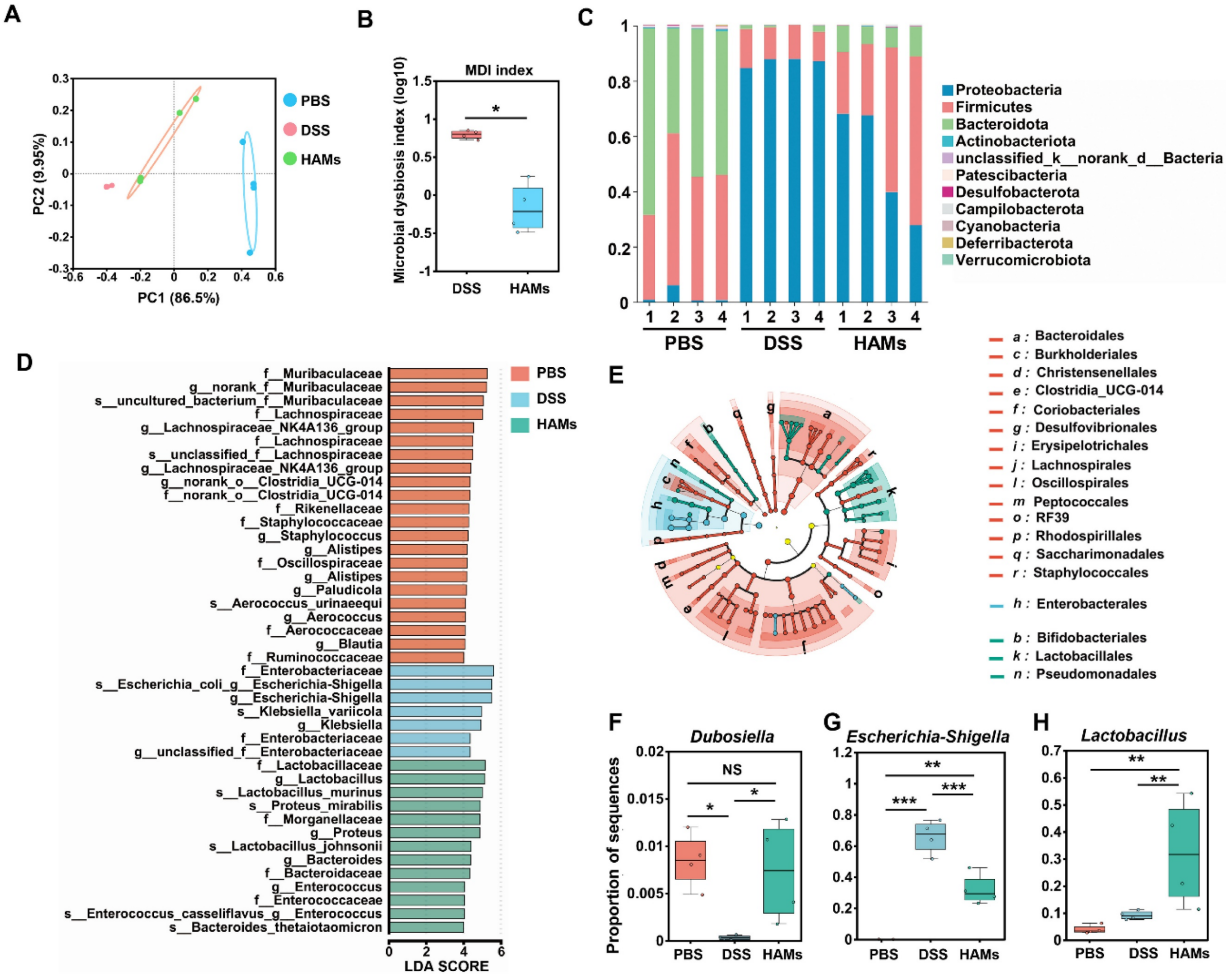
** 16S ribosomal RNA (rRNA) sequencing analysis of the gut microbiome regulated by HAM in IBD mice.** (A) Principal coordinate analysis (PCoA) showing the β diversity of the gut microbiome. Each point represents a mouse, and n = 4 for each group. The significance of clustering was determined via analysis of similarities (ANOSIM). (B) Microbial dysbiosis index (MDI) of the DSS and HAMs groups. (C) Community histogram showing the microbial compositional profiling at the phylum level. Linear discriminant analysis effect size (LEfSe) analysis (D) and LEfSe cladogram (E) of the PBS, DSS, and HAMs groups. Relative abundances of Dubosiella (F), Escherichia-Shigella (G), and Lactobacillus (H) in the PBS, DSS, and HAMs groups.

The results of the previous section suggested that HAMs may be involved in regulating the gut microbiota of IBD mice. The dysregulated gut microbiome of IBD patients results in intestinal epithelial barrier impairment and is an important cause of the occurrence and recurrence of IBD 33. Thus, we performed 16S sequencing for further investigation. As shown in Figure 7A, principal coordinate analysis (PCoA) revealed that the DSS group and HAMs group were significantly different from the PBS group at the phylum level, whereas the HAMs treatment group was closer to the PBS group, indicating that HAMs treatment could change the structural disorders of the gut microbiota in IBD patients. The microbial dysbiosis index (MDI) is an index that reflects the degree of dysbiosis of microorganisms. Our results revealed that the MDI of the HAMs treatment group was significantly lower than that of the DSS group (Figure 7B), suggesting that HAMs treatment can significantly reduce the degree of intestinal microbiota disorders.

We then analyzed the general enterobacterial composition of each sample at the phylum level. As shown in Figure 7C, the microbial compositions of the healthy control (PBS) and HAMs groups were more similar, with an apparent decrease in the relative abundance of Proteobacteria and an increase in Firmicutes and Bacteroidota in the HAMs treatment group compared with those in the DSS group. This result was consistent with previous research findings 34-36. In addition, linear discriminant analysis effect size (LEfSe) analysis also revealed typical enrichment of harmful microflora such as Escherichia-Shigella and Klebsiella in the DSS group (Figure 7D). HAMs treatment increased beneficial flora, including Lactobacillaceae, Bacteroidaceae, and Muribaculaceae (Figure 7D-E). Notably, the LEfSe Cladogram revealed that the HAMs treatment group was enriched with Bifidobacteriales (Figure 7E), a beneficial intestinal flora that can regulate intestinal flora disorders by inhibiting the propagation of harmful bacteria and modulating dendritic cell and macrophage activity 37. Furthermore, we focused on three microbial species that play important roles in maintaining intestinal health and IBD development. As shown in Figure 7F, the Dubosiella level in the HAMs group was comparable to that in the healthy group; Dubosiella is a newly defined beneficial bacterial species that improves the inflammatory bowel by remodeling the immune balance 38. In addition, the abundance of Escherichia-Shigella, a well-known harmful bacterium, significantly decreased in the HAMs treatment group (Figure 7G). In contrast, Lactobacillus, an important intestinal probiotic mentioned earlier, was significantly increased in the HAMs group (Figure 7H).

Taken together, these results indicated that HAMs could reduce the abundance of harmful bacteria involved in intestinal inflammation and promoted the enrichment of beneficial bacteria to help alleviate the degree of intestinal inflammation by optimizing the composition of the intestinal flora in mice.

## Discussion

Considering the wide range of feasible applications of DNA nanomedicines for disease treatment, in this study, we explored pure DNA-based nanomedicine for IBD treatment. This programmatically activated HAMs system was constructed from well-designed DNA hydrogels and AMs shell. The AMs shell provided a protective barrier to ensure that the HAMs could withstand the harsh gastric environment and achieve controllable release in the intestine. The DNA hydrogel increased the resistance of the anti-inflammatory kernel (TDN-ApTLR4) to nucleases and at the same time increased the electronegativity for better adsorption to IBD lesions. The AptATP-constructed DNA hydrogel allowed the release of TDN-ApTLR4 in response to the high concentration of ATP in the inflammatory microenvironment, and ApTLR4 induced TDN targeting to inflammatory immune cells and alleviated inflammation. Thus, this hydrogel was designed for tissue-microenvironment-cell hierarchical programmed activation to target the inflammation site and alleviate inflammation. Moreover, our research indicated that HAMs not only suppressed the secretion of inflammatory cytokines (TNF-α, IL-6, and IL-1β) and scavenged ROS and NO by regulating the expression of Nrf2 and HO-1 but also helped restoring the intestinal barrier and promoted the enrichment of beneficial bacteria in the intestinal flora. This design of HAMs resulted in no cytotoxicity, immunogenicity, or side effects observed after treatment of HAMs *in vitro* or *in vivo*.

Compared with traditional targeted inhibitors or biologics such as monoclonal antibodies, multitarget combination therapies had emerged as promising therapeutic strategies for IBD to reduce drug resistance and increase synergistic therapeutic effects 39. Single-target drugs such as the monoclonal antibody TNF-α had been found to lose their response to treatment in more than 30% of patients during treatment 40 and were prone to developing acquired drug resistance in long-term use 41. However, considering the possibility of an increased risk of immunogenicity and potential side effects, monoclonal antibodies were typically used alone rather than in combination with other monoclonal antibodies. In addition, traditional anti-inflammatory drugs, such as 5-aminosalicylic acid (5-ASA), might achieve therapeutic effects in the short term. However, some patients exhibited severe intolerance, and long-term use was associated with high toxicity and a risk of renal complications 42. In this study, we developed an innovative tailored treatment strategy for IBD by designing multitargeted HAMs. It combined with the anti-inflammatory effects of TDN, ApTLR4, which had TLR4 inhibitory activity, and ATP-responsive elements. Although the anti-inflammatory mechanism of TDN was unclear, studies had suggested that TDN might exert anti-inflammatory effects through multitarget synergy 43-46. In addition, ApTLR4 blocked the activation of TLR4 signaling induced by LPS stimulation. This aptamer also acted as a targeting element to improve the targeting of HAMs. HAMs were also designed to be released in response to the inflammatory environment, further increasing their specific role at the site of inflammation. Overall, these multitarget designs provided HAMs with good therapeutic efficacy, high specificity, and low toxicity, making them suitable for the treatment of IBD to the greatest extent and providing a good foundation for clinical translation.

Until now, intravenous injection had remained the main mode of administration of DNA materials for disease treatment studies. Previous studies demonstrated that some DNA materials combined with other materials could affect cell biological behaviors and inhibited the development of cancer through intravenous injection 47. DNA materials had also been reported to be good drug carriers for enhancing drug targeting efficiency in various disease treatments. Compared with conventional nanoscale drug carriers (gold nanoparticles, metal‒organic frameworks, liposomes, etc.), DNA materials were more suitable candidates because of their unique features, including high biocompatibility, astructural programmability, low cytotoxicity, and ability to penetrate cell membranes without transfection. However, some limitations of DNA materials, such as the short blood circulation time and poor aggregation in target tissues, remained to overcome before their use in the clinic 48. Therefore, special administration methods for specific diseases might effectively overcome these limitations. Rui Ye et al. reported a DNAgel network for accelerating wound healing by applying it to the wound skin area. The network had specific characteristics for rapid water absorption, high swelling, and instant tissue adhesion to physically stop bleeding 49. In addition, Yuting Xie et al reported that a quantitative nebulized drug delivery for TDN carried pirfenidone to the lungs for lung fibrosis treatment 50. These special administration methods could greatly increase the local concentration of the drug and reduce side effects. In this study, we conducted an oral administration of DNA hydrogel coated with AMs for IBD treatment. Even in the long-term toxicology evaluation, we did not observe abnormal phenotypes, apparent tissue damage or severe side effects in the liver, spleen, lung or kidney. Thus, this administration method could greatly reduce side effects and improve therapeutic effects by increasing the local drug concentration and reducing the number of times and dosage of drug administered. Compared with systemic administration, this topical administration achieved a more effective and safer treatment effect.

Dysbiosis of the gut microbiota played a critical role in the development of IBD and had been demonstrated to influence the effectiveness of treatments. Many drugs or novel materials had been reported to affect the composition of the gut microbiota. Wang, M. et al. reported that an oral pifithrin-α-embedded nanomedicine effectively treats IBD by inhibiting excessive IEC apoptosis 51. This nanomedicine inhibited harmful bacteria and increased beneficial bacteria by effectively disrupting the vicious cycle of harmful bacterial colonization and inflammatory response activation 51. From the perspective of inhibiting inflammation, a triangular DNA origami nanostructure loaded with siTNFα (^siTNF-α^ tDON) was designed and found to effectively mitigate inflammation 52. ^siTNF-α^ tDON was also demonstrated to effectively restore gut microbiota abundance and increase the relative prevalence of beneficial bacteria to reinstate gut homeostasis 52. In this work, we found that HAMs could reduce the abundance of harmful bacteria involved in intestinal inflammation and promote the enrichment of beneficial bacteria. Therefore, the degree of intestinal inflammation could be alleviated by optimizing the composition of the intestinal flora in mice. However, there had been no reports showing that DNA nanomaterials have a direct effect on bacteria. We hypothesized that the regulation of the intestinal microbiota composition by HAMs might also be indirectly influenced by the alleviation of host inflammation. Moreover, here, we had only studied the effects of short-term treatment on the gut microbiota, which seemed to limit the extent of microbial changes, and further research was needed to explore the impact of long-term treatment on the microbiota.

## Conclusions

In summary, this pure DNA-based microcapsule system demonstrated high-precision targeting and excellent anti-inflammatory therapeutic effects in an IBD mouse model, suggesting its potential as a nanomedicine for IBD treatment. More importantly, this HAMs system represented the first instance of the oral targeted administration of DNA nanomedicine, providing new insights into their comprehensive application in disease treatment and suggesting exciting possibilities in the field of nanomedicine.

## Supplementary Material

Supplementary figures and tables.

## Abbreviations

HAMs: DNA hydrogel microcapsules
IBD: inflammatory bowel disease
AMs: sodium alginate microcapsules
TDN: tetrahedral DNA nanostructure
DSS: dextran sodium sulfate
TNF-α: tumor necrosis factor-α
LPS: lipopolysaccharide
ApTLR4: TLR4 aptamer
TLR4: toll-like receptor 4
SGF: simulated gastric fluid
SIF: simulated intestinal fluid
TDN@AMs: AMs coated TDN
ApTLR4@AMs: AMs coated ApTLR4
DAMs: AMs coated TDN-ApTLR4
HAMs: AMs coated DNA hydrogel
DAI: disease activity index
MDI: microbial dysbiosis index

## References

[1] Bisgaard TH, Allin KH, Keefer L, Ananthakrishnan AN, Jess T (2022). Depression and anxiety in inflammatory bowel disease: epidemiology, mechanisms and treatment. Nat Rev Gastroenterol Hepatol.

[2] Danne C, Skerniskyte J, Marteyn B, Sokol H (2024). Neutrophils: from IBD to the gut microbiota. Nat Rev Gastroenterol Hepatol.

[3] Kotla NG, Rochev Y (2023). IBD disease-modifying therapies: insights from emerging therapeutics. Trends Mol Med.

[4] Pan Y, Zhang H, Li M, He T, Guo S, Zhu L (2024). Novel approaches in IBD therapy: targeting the gut microbiota-bile acid axis. Gut Microbes.

[5] Heller C, Moss AC, Rubin DT (2024). Overview to Challenges in IBD 2024-2029. Inflamm Bowel Dis.

[6] Abed OA, Attlassy Y, Xu J, Han K, Moon JJ (2022). Emerging Nanotechnologies and Microbiome Engineering for the Treatment of Inflammatory Bowel Disease. Mol Pharm.

[7] Tian T, Zhang T, Shi S, Gao Y, Cai X, Lin Y (2023). A dynamic DNA tetrahedron framework for active targeting. Nat Protoc.

[8] Chhabra R, Sharma J, Liu Y, Rinker S, Yan H (2010). DNA self-assembly for nanomedicine. Adv Drug Deliv Rev.

[9] Tian R, Shang Y, Wang Y, Jiang Q, Ding B (2023). DNA Nanomaterials-Based Platforms for Cancer Immunotherapy. Small Methods.

[10] Mendes BB, Conniot J, Avital A, Yao D, Jiang X, Zhou X (2022). Nanodelivery of nucleic acids. Nat Rev Methods Primers.

[11] Heuer-Jungemann A, Linko V (2021). Engineering Inorganic Materials with DNA Nanostructures. ACS Cent Sci.

[12] Dovgan I, Koniev O, Kolodych S, Wagner A (2019). Antibody-Oligonucleotide Conjugates as Therapeutic, Imaging, and Detection Agents. Bioconjug Chem.

[13] Zhou M, Gao S, Zhang X, Zhang T, Zhang T, Tian T (2021). The protective effect of tetrahedral framework nucleic acids on periodontium under inflammatory conditions. Bioact Mater.

[14] Gao S, Zhou M, Li Y, Xiao D, Wang Y, Yao Y (2021). Tetrahedral Framework Nucleic Acids Reverse New-Onset Type 1 Diabetes. ACS Appl Mater Interfaces.

[15] Zhang Q, Lin S, Wang L, Peng S, Tian T, Li S (2021). Tetrahedral framework nucleic acids act as antioxidants in acute kidney injury treatment. Chemical Engineering Journal.

[16] Gao S, Wang Y, Li Y, Xiao D, Lin Y, Chen Y (2021). Tetrahedral Framework Nucleic Acids Reestablish Immune Tolerance and Restore Saliva Secretion in a Sjogren's Syndrome Mouse Model. ACS Appl Mater Interfaces.

[17] Zhou M, Liu NX, Shi SR, Li Y, Zhang Q, Ma QQ (2018). Effect of tetrahedral DNA nanostructures on proliferation and osteo/odontogenic differentiation of dental pulp stem cells via activation of the notch signaling pathway. Nanomedicine.

[18] Zhang Q, Lin S, Shi S, Zhang T, Ma Q, Tian T (2018). Anti-inflammatory and Antioxidative Effects of Tetrahedral DNA Nanostructures via the Modulation of Macrophage Responses. ACS Appl Mater Interfaces.

[19] Liu J, Kang R, Tang D (2024). Lipopolysaccharide delivery systems in innate immunity. Trends Immunol.

[20] Stephens M (2022). The emerging potential of Aptamers as therapeutic agents in infection and inflammation. Pharmacology & Therapeutics.

[21] Dosch M, Gerber J, Jebbawi F, Beldi G (2018). Mechanisms of ATP Release by Inflammatory Cells. Int J Mol Sci.

[22] Li F, Lyu D, Liu S, Guo W (2020). DNA Hydrogels and Microgels for Biosensing and Biomedical Applications. Adv Mater.

[23] Wei Y, Wang K, Luo S, Li F, Zuo X, Fan C (2022). Programmable DNA Hydrogels as Artificial Extracellular Matrix. Small.

[24] Lee Y, Kamada N, Moon JJ (2021). Oral nanomedicine for modulating immunity, intestinal barrier functions, and gut microbiome. Adv Drug Deliv Rev.

[25] Zhang T, Tian T, Zhou R, Li S, Ma W, Zhang Y (2020). Design, fabrication and applications of tetrahedral DNA nanostructure-based multifunctional complexes in drug delivery and biomedical treatment. Nat Protoc.

[26] Fernandez G, Moraga A, Cuartero MI, Garcia-Culebras A, Pena-Martinez C, Pradillo JM (2018). TLR4-Binding DNA Aptamers Show a Protective Effect against Acute Stroke in Animal Models. Mol Ther.

[27] Shivaji UN, Nardone OM, Cannatelli R, Smith SC, Ghosh S, Iacucci M (2020). Small molecule oral targeted therapies in ulcerative colitis. Lancet Gastroenterol Hepatol.

[28] Gheorghita R, Sirbu IO, Lobiuc A, Covasa M (2024). Sodium Alginate-Starch Capsules for Enhanced Stability of Metformin in Simulated Gastrointestinal Fluids. Biomimetics (Basel).

[29] Ciarleglio G, Placido M, Toto E, Santonicola MG (2024). Dual-Responsive Alginate/PNIPAM Microspheres Fabricated by Microemulsion-Based Electrospray. Polymers (Basel).

[30] Uyen NTT, Hamid ZAA, Tram NXT, Ahmad N (2020). Fabrication of alginate microspheres for drug delivery: A review. Int J Biol Macromol.

[31] Luo R, Liu J, Cheng Q, Shionoya M, Gao C, Wang R (2024). Oral microsphere formulation of M2 macrophage-mimetic Janus nanomotor for targeted therapy of ulcerative colitis. Sci Adv.

[32] Zhang Y, Li X, Luo Z, Ma L, Zhu S, Wang Z (2020). ECM1 is an essential factor for the determination of M1 macrophage polarization in IBD in response to LPS stimulation. Proc Natl Acad Sci U S A.

[33] Shan Y, Lee M, Chang EB (2022). The Gut Microbiome and Inflammatory Bowel Diseases. Annu Rev Med.

[34] Sun D, Bai R, Zhou W, Yao Z, Liu Y, Tang S (2021). Angiogenin maintains gut microbe homeostasis by balancing α-Proteobacteria and Lachnospiraceae. Gut.

[35] Wang X, Cai Z, Wang Q, Wu C, Sun Y, Wang Z (2024). Bacteroides methylmalonyl-CoA mutase produces propionate that promotes intestinal goblet cell differentiation and homeostasis. Cell Host Microbe.

[36] Martin R, Rios-Covian D, Huillet E, Auger S, Khazaal S, Bermudez-Humaran LG (2023). Faecalibacterium: a bacterial genus with promising human health applications. FEMS Microbiol Rev.

[37] Gavzy SJ, Kensiski A, Lee ZL, Mongodin EF, Ma B, Bromberg JS (2023). Bifidobacterium mechanisms of immune modulation and tolerance. Gut Microbes.

[38] Zhang Y, Tu S, Ji X, Wu J, Meng J, Gao J (2024). Dubosiella newyorkensis modulates immune tolerance in colitis via the L-lysine-activated AhR-IDO1-Kyn pathway. Nat Commun.

[39] Battat R, Chang JT, Loftus EV Jr, Sands BE (2025). IBD Matchmaking: Rational Combination Therapy. Clin Gastroenterol Hepatol.

[40] Schmitt H, Billmeier U, Dieterich W, Rath T, Sonnewald S, Reid S (2019). Expansion of IL-23 receptor bearing TNFR2+ T cells is associated with molecular resistance to anti-TNF therapy in Crohn's disease. Gut.

[41] Sidiropoulos PI, Boumpas DT (2006). Differential drug resistance to anti-tumour necrosis factor agents in rheumatoid arthritis. Ann Rheum Dis.

[42] Mikami Y, Tsunoda J, Suzuki S, Mizushima I, Kiyohara H, Kanai T (2023). Significance of 5-Aminosalicylic Acid Intolerance in the Clinical Management of Ulcerative Colitis. Digestion.

[43] Jiang Y, Li S, Zhang T, Zhang M, Chen Y, Wu Y (2022). Tetrahedral Framework Nucleic Acids Inhibit Skin Fibrosis via the Pyroptosis Pathway. ACS Applied Materials & Interfaces.

[44] Wang J, Xie N, Lu S, Xiao B, Ou Y, Li Q (2024). CpG and Rutin Co-Loaded DNA Tetrahedra for Targeted Therapy of Intracerebral Hemorrhage: Synergistic Hematoma Clearance and Neuroinflammation Inhibition. Advanced Functional Materials.

[45] Chen X, He J, Xie Y, Zhang T, Li S, Zhao Y (2023). Tetrahedral framework nucleic acid nanomaterials reduce the inflammatory damage in sepsis by inhibiting pyroptosis. Cell Prolif.

[46] Wang L, Wang Y, Jiang Y, Chen M, Li Z, Wang K (2023). Tetrahedral Framework Nuclear Acids Can Regulate Interleukin-17 Pathway to Alleviate Inflammation and Inhibit Heterotopic Ossification in Ankylosing Spondylitis. ACS Nano.

[47] Li C, Wang M, Li PF, Sheng J, Fu Q (2024). Construction of Smart DNA-Based Drug Delivery Systems for Cancer Therapy. Small.

[48] Ma W, Zhan Y, Zhang Y, Mao C, Xie X, Lin Y (2021). The biological applications of DNA nanomaterials: current challenges and future directions. Signal Transduction and Targeted Therapy.

[49] Ye R, Zhu Z, Gu T, Cao D, Jiang K, Dai Q (2024). Neutrophil extracellular traps-inspired DNA hydrogel for wound hemostatic adjuvant. Nat Commun.

[50] Xie Y, Shi S, Lv W, Wang X, Yue L, Deng C (2024). Tetrahedral Framework Nucleic Acids Delivery of Pirfenidone for Anti-Inflammatory and Antioxidative Effects to Treat Idiopathic Pulmonary Fibrosis. ACS Nano.

[51] Wang M, Huang Q, Liu M, Zhao T, Song X, Chen Q (2023). Precisely Inhibiting Excessive Intestinal Epithelial Cell Apoptosis to Efficiently Treat Inflammatory Bowel Disease with Oral Pifithrin-alpha Embedded Nanomedicine (OPEN). Adv Mater.

[52] Ge J, Jia B, Wang Y, Ma Y, Sun X, Dong J (2024). DNA Nanostructures Treat Inflammatory Bowel Disease through ROS Scavenging and Gut Microbiota Modulation. Advanced Functional Materials.

