# Supplementary material for: Programmatically activated DNA hydrogel microcapsules for precision therapy in inflammatory bowel disease
Source: Theranostics. 2025 May 8;15(13):6203–20. doi: 10.7150/thno.111583 (PMC12159827; doi:10.7150/thno.111583)
Supplement: Supplementary file 1 — Supplementary figures and tables. [file thnov15p6203s1.pdf]

Supporting Information

Programmatically activated DNA hydrogel microcapsules for precision therapy in inflammatory bowel disease

Peifen Lu[1,#], Hongxiu Yuan[1,#], Gang Wang[1], Yixi Dong[1], Runyu Zhao[1], Jia Man[2], Jianwei Jiao[3,], Zhaoyin Wang[4,*], Jin Jiao[1,*]*

Table of Contents

1. Supplementary tables

Table S1 Oligonucleotide sequences used in this work..3

Table S2 Primer sequences for RT-qPCR analysis..5

2. Supplementary Figures

Figure S1-5 Characterizations of DNA hydrogel...6

Figure S6 Biocompatibility of DNA hydrogel...11

Figure S7 Anti-inflammatory effect of DNA hydrogel..12

Figure S8 Scratch wound healing of Caco2...13

Figure S9 Anti-inflammatory effect of DNA hydrogel in Caco2……………………………………14

Figure S10 The robust anti-inflammatory effect of DNA hydrogel. 15

Figure S11 Characterizations of sodium alginate microspheres...16

Figure S12 Stability of TDN-ApTLR4 and TDN hydrogel in SIF...17

Figure S13 Therapeutic efficacy of DNA hydrogel on IBD model mice.......................................18

Figure S14 Hematoxylin and eosin (H&E) staining of the major organs of IBD or healthy control mice under different treatment……………………………………………………………...19

Figure S15-17 Safety evaluation of HAMs……………………………………………………...22

Figure S18 mRNA sequencing of intestinal tissues in IBD mice after treatment with HAMs...23

Table S1. Oligonucleotide sequences used in this work.

**Name**	**Sequence (5'-3')**
S1-1	ATTTATCACCCGCCATAGTAGACGTATCACCAGGCAGTTGAGACGAACATTCCTAAGTCTGAATTTTTT**CCCCCAGGTTCTCT**
S1-2	ATTTATCACCCGCCATAGTAGACGTATCACCAGGCAGTTGAGACGAACATTCCTAAGTCTGAATTTTTT**CCCCCAGGTTCT**
S2-2	ACATGCGAGGGTCCAATACCGACGATTACAGCTTGCTACACGATTCAGACTTAGGAATGTTCGTTTTTT**CCCCCAGGTTCT**
S3-2	ACTACTATGGCGGGTGATAAACGTGTAGCAAGCTGTAATCGACGGGAAGAGCATGCCCATCCTTTTTT**CCCCCAGGTTCT**
S4	GGTGTGCCAATAAACCATATCGCCGCGTTAGCATGTACTCGGTTGGCCCTAAATACGAGTTTTTTTTACGGTATTGGACCCTCGCATGACTCAACTGCCTGGTGATACGAGGATGGGCATGCTCTTCCCG
TDN-S4	ACGGTATTGGACCCTCGCATGACTCAACTGCCTGGTGATACGAGGATGGGCATGCTCTTCCCG
Y1-1	CACGCTGTCCTAACCATGACCGTCGAAG**AGAGAACCTGGGGG**AGTATTGCGGAGGAAGGT
Y1-2	CACGCTGTCCTAACCATGACCGTCGAAG**AGAACCTGGGGG**AGTATTGCGGAGGAAGGT
Y2-2	CTTCGACGGTCATGTACTAGATCAGAGG**AGAACCTGGGGG**AGTATTGCGGAGGAAGGT
Y3-2	CCTCTGATCTAGTAGTTAGGACAGCGTG**AGAACCTGGGGG**AGTATTGCGGAGGAAGGT
S1-2-FAM	ATTTATCACCCGCCATAGTAGACGTATCACCAGGCAGTTGAGACGAACATTCCTAAGTCTGAATTTTTT**CCCCCAGGTTCT**-FAM
Y2-2-BHQ1	CTTCGACGGTCATG/dT-BHQ1/ACTAGATCAGAGG**AGAACCTGGGGG**AGTATTGCGGAGGAAGGT
ApTLR4	GGTGTGCCAATAAACCATATCGCCGCGTTAGCATGTACTCGGTTGGCCCTAAATACGAG

FAM-ApTLR4	FAM-GGTGTGCCAATAAACCATATCGCCGCGTTAGCATGTACTCGGTTGGCCCTAAATACGAG

Note: Bold region represents the hydridized part of "TDN" and "Y Scrafford". Blue and red region indicate ATP apamer (Apt_{ATP}) and TLR4 apamer (ApTLR4), respectively.

Table S2. Primer sequences for RT-qPCR analysis

Gene	Forward Primer(5’-3’)	Reverse Primer(5’-3’)
ACTB	GTGACGTTGACATCCGTAAAGA	GCCGGACTCATCGTACTCC
TNF-α	CAGGCGGTGCCTATGTCTC	CGATCACCCCGAAGTTCAGTAG
IL-6	CTGCAAGAGACTTCCATCCAG	AGTGGTATAGACAGGTCTGTTGG
IL-1β	GAAATGCCACCTTTTGACAGTG	TGGATGCTCTCATCAGGACAG
iNOS	GTTCTCAGCCCAACAATACAAGA	GTGGACGGGTCGATGTCAC
HO-1	AGGTACACATCCAAGCCGAGA	CATCACCAGCTTAAAGCCTTCT
Occludin	ACAAGCGGTTTTATCCAGAGTC	GTCATCCACAGGCGAAGTTAAT
Claudin1	CCTCCTGGGAGTGATAGCAAT	GGCAACTAAAATAGCCAGACCT

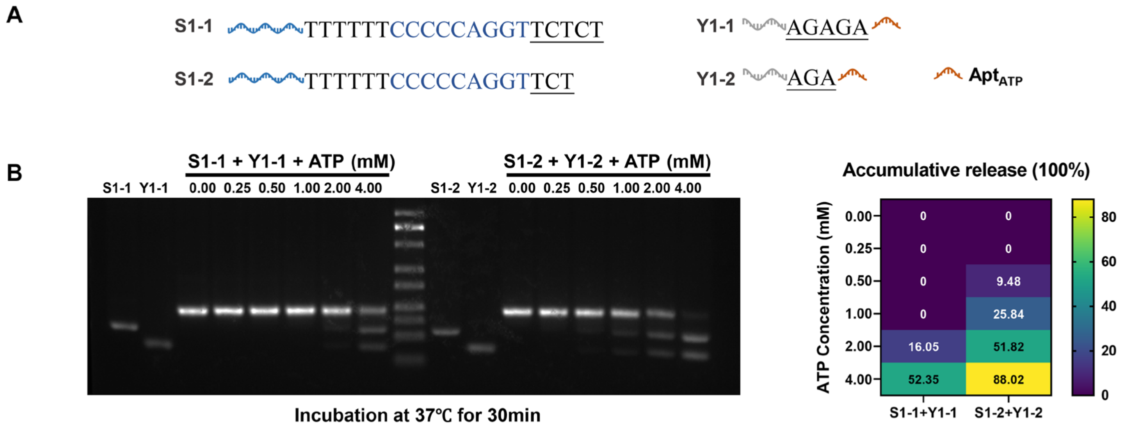

Figure S1 Characterizations of DNA hydrogel. (A) Schematic diagram of complementary bases number of "TDN" and "Y Scrafford". Different numbers of optimized bases are highlighted with underlining. (B) Agarose gel electrophoresis of the ATP inducing unraveled of hybrid strands and its gray scale statistical analysis.

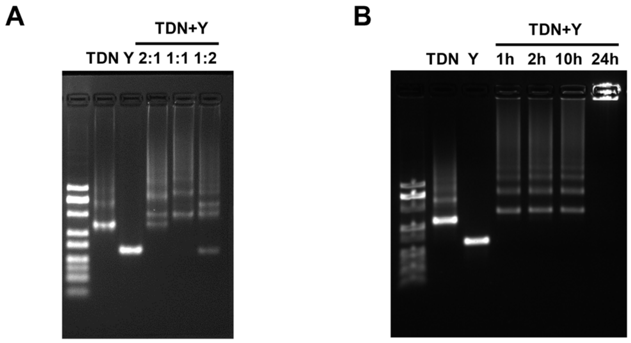

Figure S2 Characterizations of DNA hydrogel. Agarose gel electrophoresis of TDN-ApTLR4 and "Y Scrafford" assembling under different mix ratio (A) and incubation time (B).

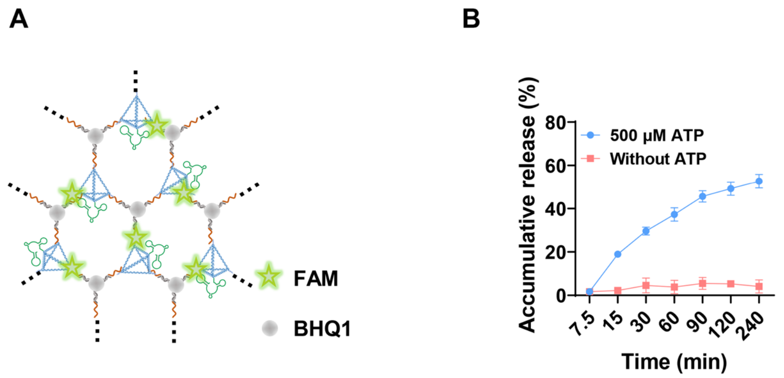

Figure S3 Characterizations of DNA hydrogel. (A) Diagram of FAM / BHQ1 labeled TDN hydrogel. (B) Release rate of DNA hydrogel responded to ATP at pH 6.5.

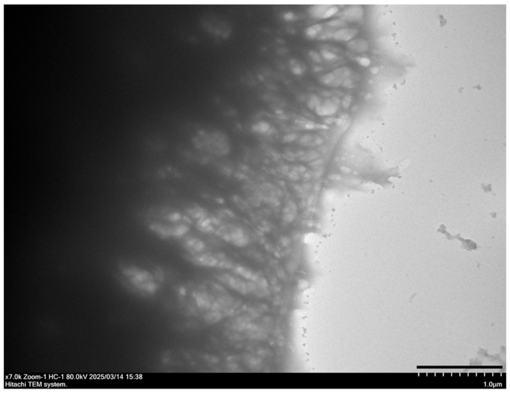

Figure S4 Characterizations of DNA hydrogel. The TEM images of the DNA hydrogel.

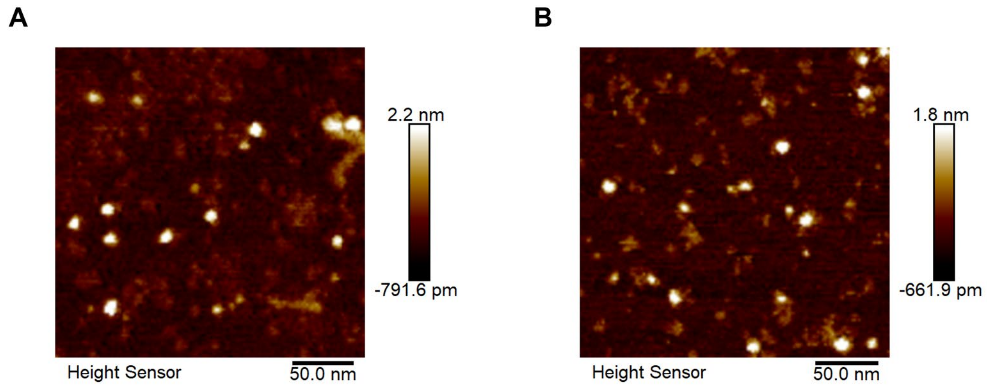

Figure S5 Characterizations of DNA hydrogel. The AFM images of the TDN-ApTLR4 (A) and Y-AptATP scaffold (B).

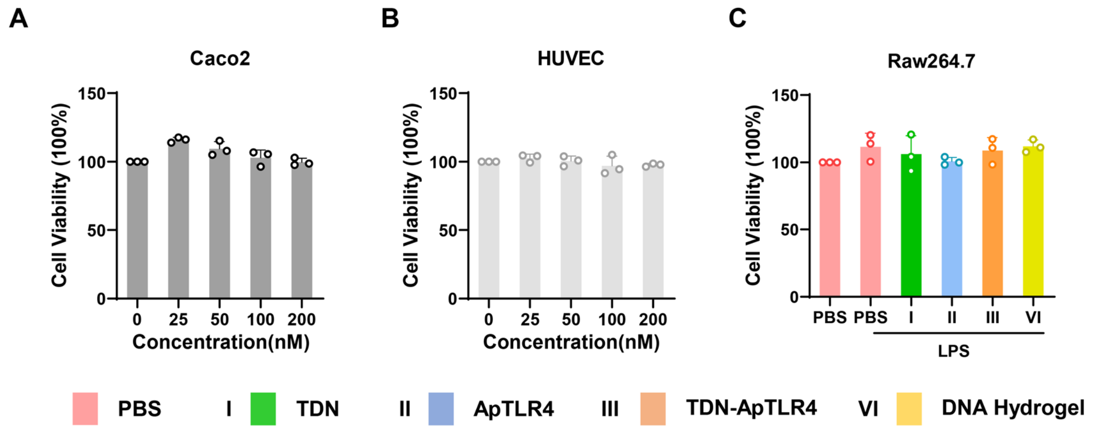

Figure S6 Biocompatibility of DNA hydrogel. Biocompatibility of various concentrations of TDN hydrogel toward Caco2 (A) and HUVEC (B). (C) Biocompatibility of different nanostructures toward RAW264.7 cells under stimulating of LPS.

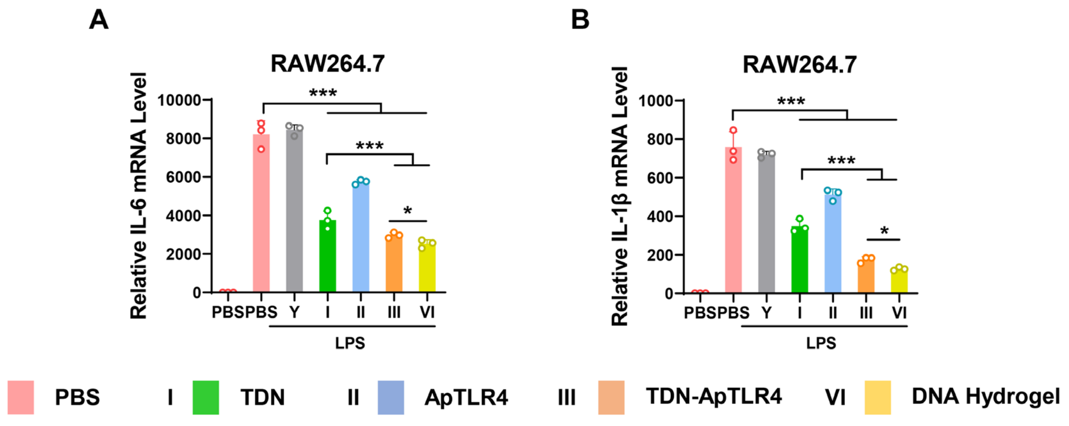

Figure S7 Anti-inflammatory effect of DNA hydrogel. mRNA expression of IL-6 (A) and IL-1β (B) in RAW264.7 after different treatments. Y, Y-AptATP scaffold.

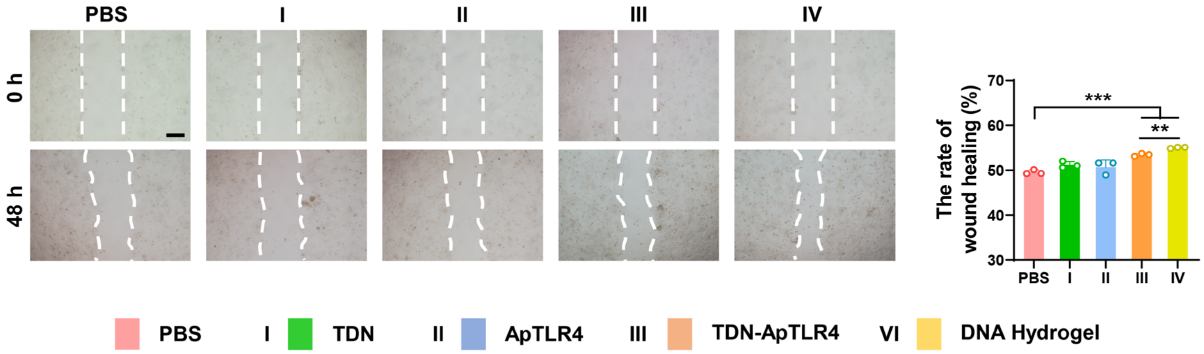

Figure S8 Scratch wound healing of Caco2 under different treatments.

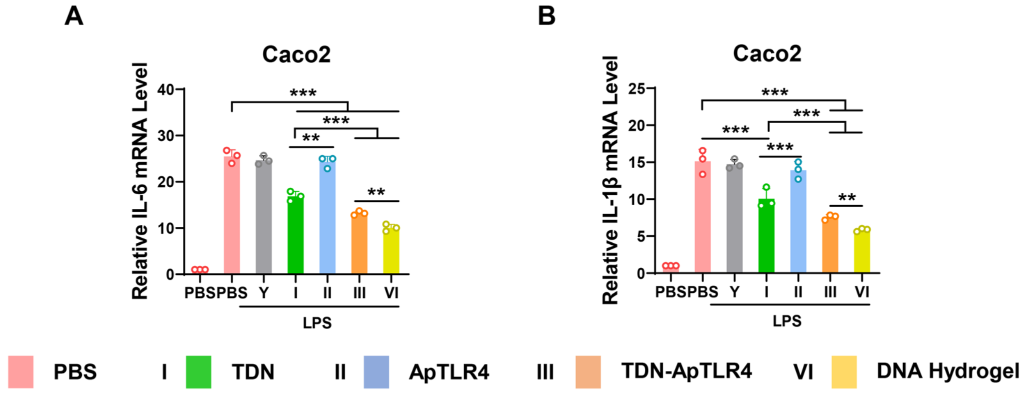

Figure S9 Anti-inflammatory effect of DNA hydrogel in Caco2.

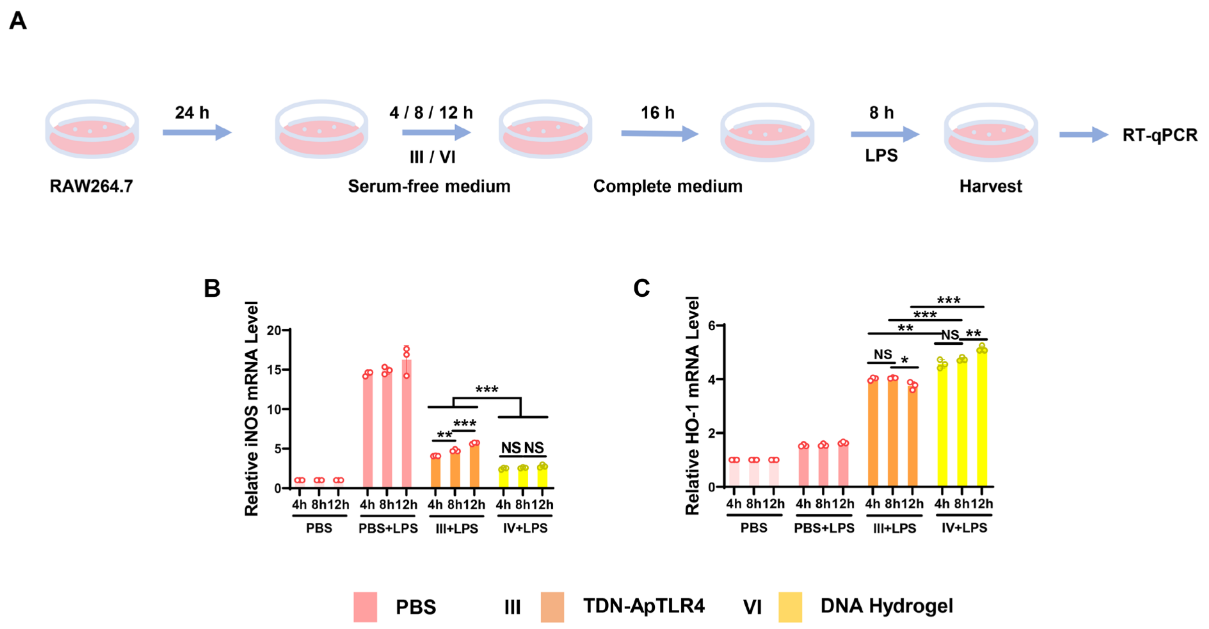

Figure S10 The robust anti-inflammatory effect of DNA hydrogel. (A) Diagram of experimental procedures. mRNA expression of iNOS (B) and HO-1 (C) in RAW264.7 after different treatments under increasing treatment time.

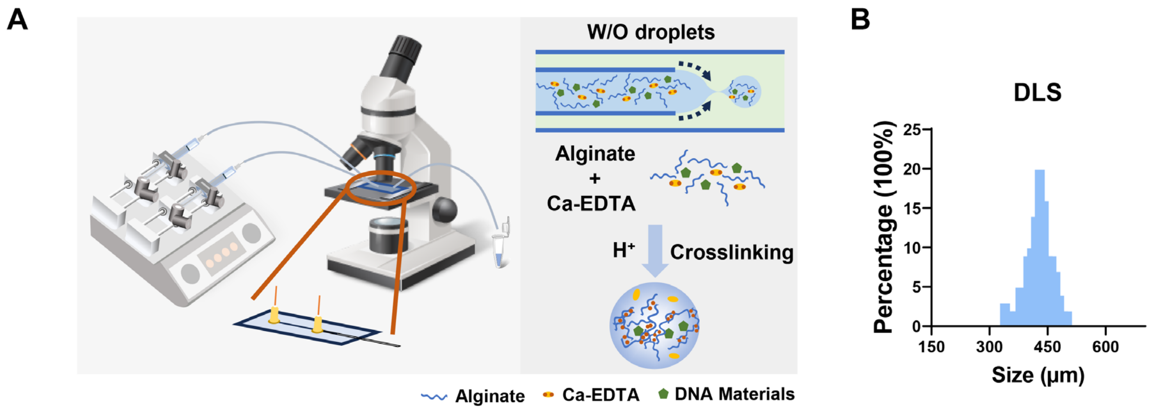

Figure S11 Characterizations of sodium alginate microspheres. (A) Schematic diagram of sodium alginate microspheres (AMs). (B) DLS of AMs.

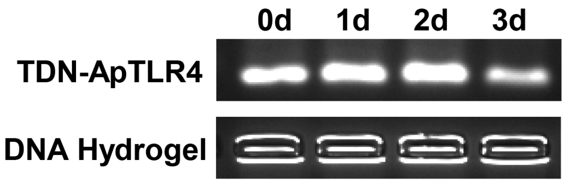

Figure S12 Stability of TDN-ApTLR4 and TDN hydrogel in SIF. Agarose gel electrophoresis of TDN-ApTLR4 and TDN hydrogel in different time incubation with SIF. SIF, simulated intestinal fluid.

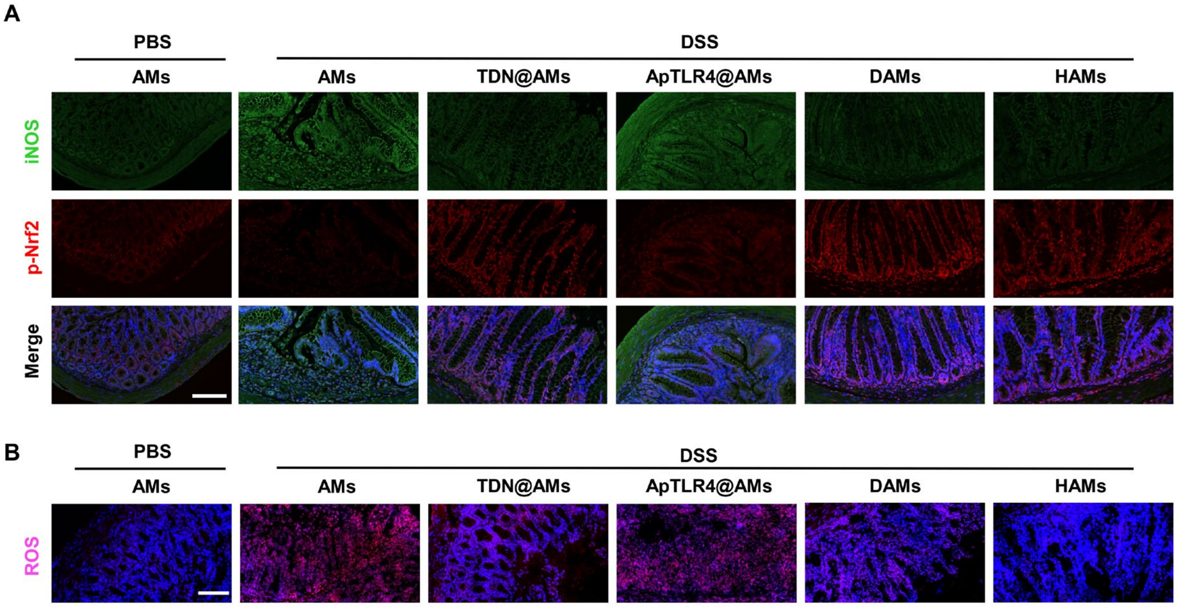

Figure S13 Therapeutic efficacy of DNA hydrogel on IBD model mice. (A) Immunofluorescence staining of the two key molecules of NO production and ROS metabolic pathway, iNOS and Nrf2 of colons of IBD or healthy control mice under different treatment. Scale bar, 100 μm. (B) ROS level confirmed by dihydroethidium (DHE) staining of colons of IBD or healthy control mice under different treatment. Scale bar, 100 μm.

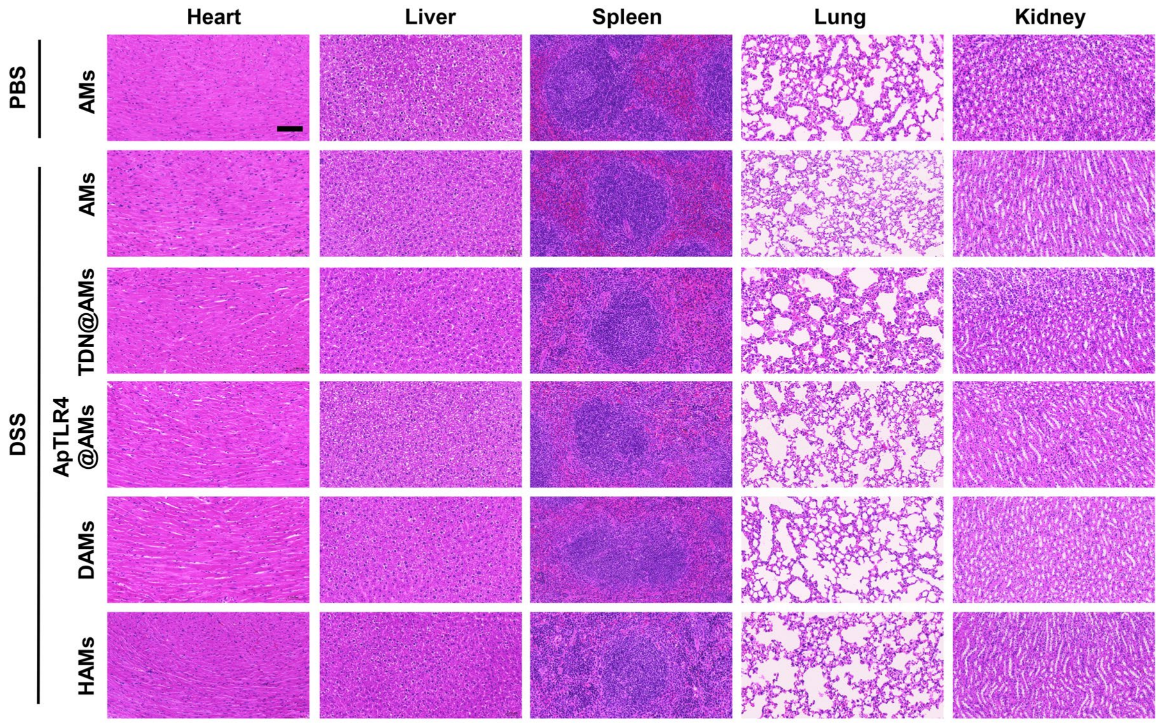

Figure S14 Hematoxylin and eosin (H&E) staining of the major organs of IBD or healthy control mice under different treatment. Scale bar, 50 μm.

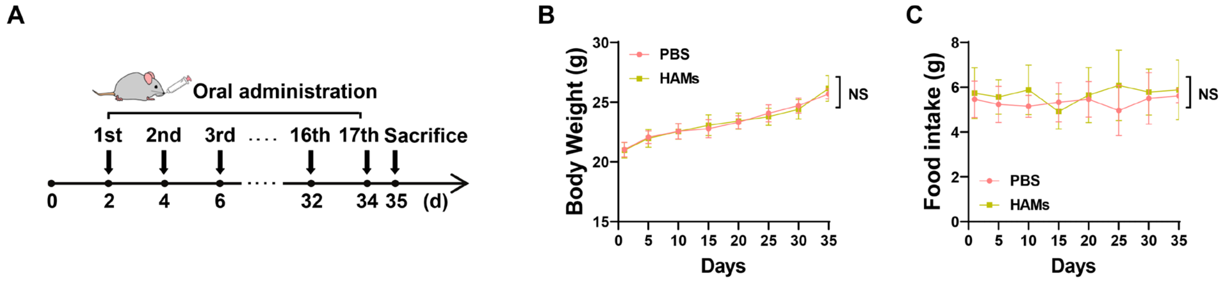

Figure S15 Safety evaluation of HAMs. (A) Schematic illustration of the experimental design. Changes in mouse body weight (B) and food intake (C) after long-term HAMs treatment.

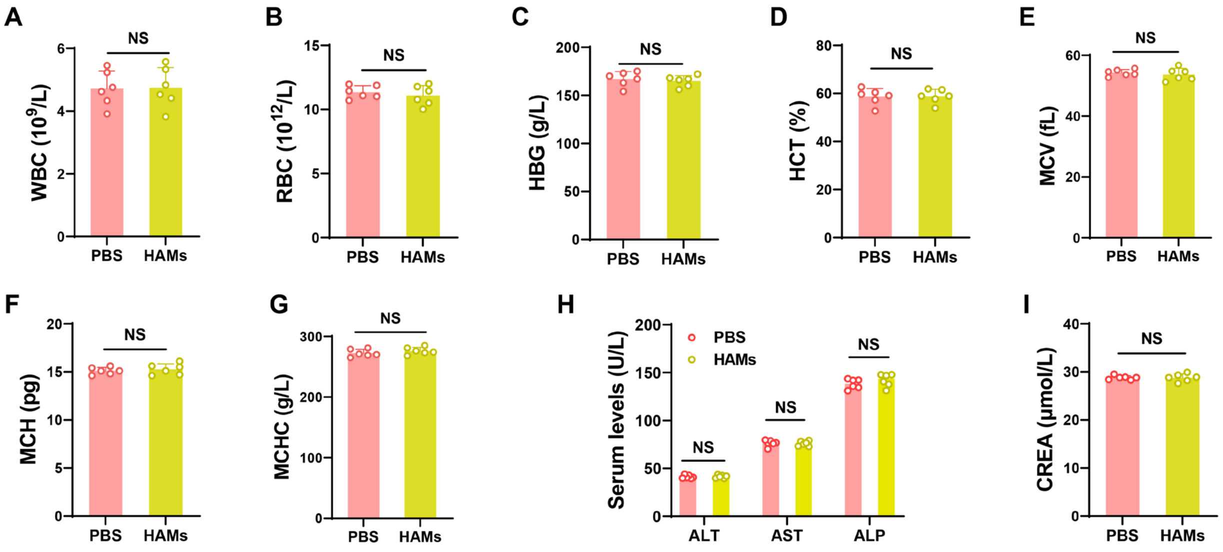

Figure S16 Safety evaluation of HAMs. (A-G) Blood biochemical tests of white blood cells (WBCs), red blood cells (RBCs), hemoglobin (HGB), hematocrit (HCT), mean corpuscular volume (MCV), mean corpuscular hemoglobin (MCH), and mean corpuscular hemoglobin concentration (MCHC) after long-term HAMs treatment. (H-I) Changes in the cytokine response of glutamic pyruvic transaminase (ALT), glutamic oxaloacetic transaminase (AST), alkaline phosphatase (ALP) and creatinine (CREA) in mice after long-term HAM treatment.

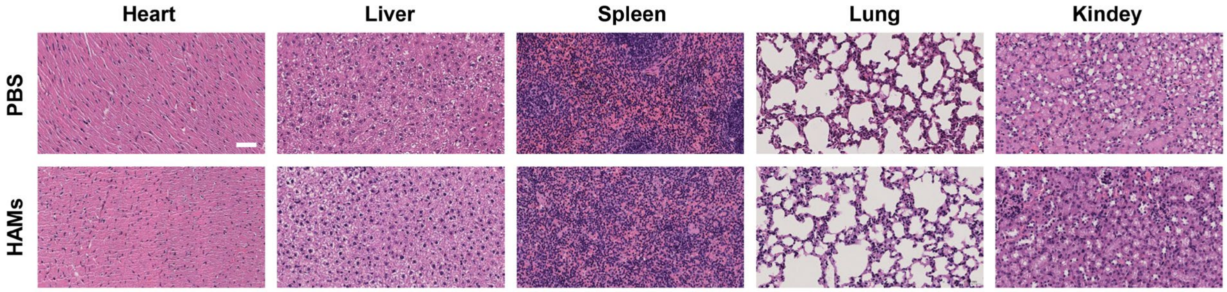

Figure S17 Safety evaluation of HAMs. Histological images of the main organs after treatment. Scale bar: 50 μm.

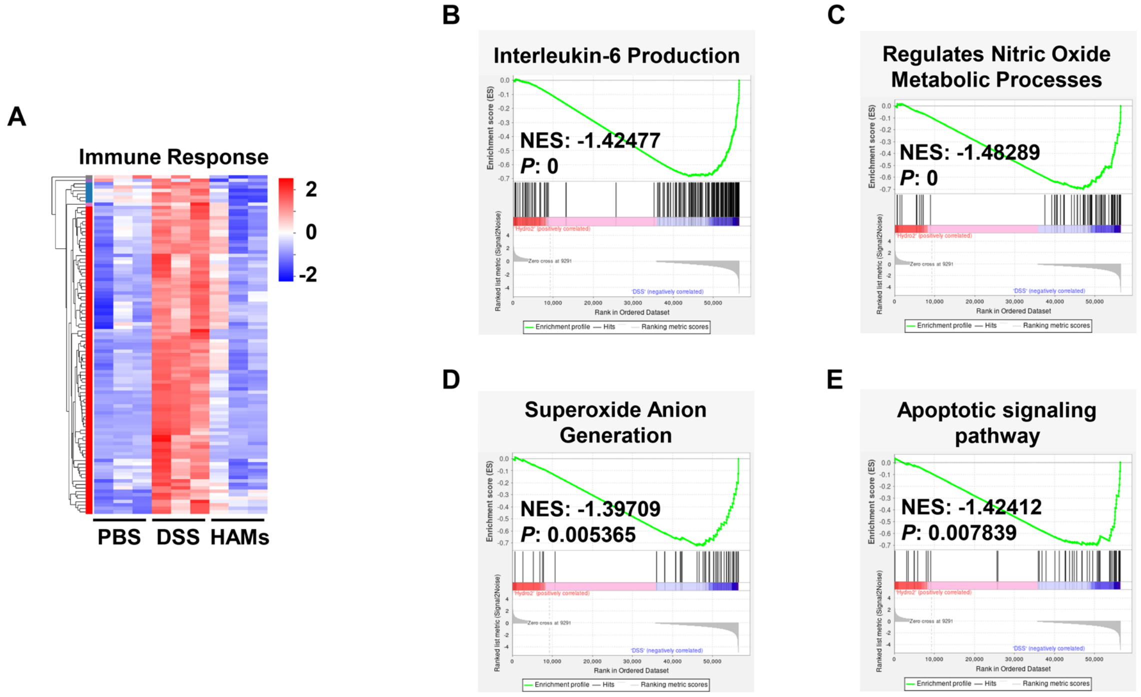

Figure S18 mRNA sequencing of intestinal tissues in IBD mice after treatment with HAMs. (A) Heat map of markers for immune response in PBS, DSS, and HAMs groups. Enrichment plots from GSEA analyses of genesets for "Interleukin-6 Production" (B), "Regulates Nitric Oxide Metabolic Processes" (C), "Superoxide Anion Generation" (D), and "Apoptotic signaling pathway" (E). NES, normalized enrichment score. $|NES| > 1$, p-value < 0.05 were considered statistically significant in PBS versus HAMs groups.
